# Tuning structural modulation and magnetic properties in metal–organic coordination polymers [CH_3_NH_3_]Co_*x*_Ni_1−*x*_(HCOO)_3_

**DOI:** 10.1107/S2052252524008583

**Published:** 2024-09-24

**Authors:** Madeleine Geers, Oscar Fabelo, Matthew J. Cliffe, Laura Cañadillas-Delgado

**Affiliations:** a Diffraction Group Institut Laue Langevin 71 avenue des Martyrs Grenoble38042 France; b School of Chemistry, University Park, NottinghamNG7 2RD, United Kingdom; Formby, Liverpool, United Kingdom

**Keywords:** phase transitions, incommensurate structures, solid solutions, formate ligands, aperiodic structures, materials science, magnetic structures, coordination polymers

## Abstract

Three solid solutions of [CH_3_NH_3_]Co*_x_*Ni_1−*x*_(HCOO)_3_, with *x* = 0.25 (**1**), *x* = 0.50 (**2**) and *x* = 0.75 (**3**), were synthesized and their nuclear structures and magnetic properties were characterized using single-crystal neutron diffraction and magnetization measurements. At room temperature, all three compounds crystallize in the *Pnma* orthorhombic space group, akin to the cobalt and nickel end series members. On cooling, each compound undergoes a distinct series of structural transitions to modulated structures. Compound **1** exhibits a phase transition to a modulated structure analogous to the pure Ni compound [Cañadillas-Delgado, L., Mazzuca, L., Fabelo, O., Rodríguez-Carvajal, J. & Petricek, V. (2020). *Inorg. Chem.***59**, 17896–17905], whereas compound **3** maintains the behaviour observed in the pure Co compound reported previously [Canadillas-Delgado, L., Mazzuca, L., Fabelo, O., Rodriguez-Velamazan, J. A. & Rodriguez-Carvajal, J. (2019). *IUCrJ*, **6**, 105–115], although in both cases the temperatures at which the phase transitions occur differ slightly from the pure phases. Monochromatic neutron diffraction measurements showed that the structural evolution of **2** diverges from that of either parent compound, with competing hydrogen bond interactions that drive the modulation throughout the series, producing a unique sequence of phases. It involves two modulated phases below 96 (3) and 59 (3) K, with different **q** vectors, similar to the pure Co compound (with modulated phases below 128 and 96 K); however, it maintains the modulated phase below magnetic order [at 22.5 (7) K], resembling the pure Ni compound (which presents magnetic order below 34 K), resulting in an improper modulated magnetic structure. Despite these large-scale structural changes, magnetometry data reveal that the bulk magnetic properties of these solid solutions form a linear continuum between the end members. Notably, doping of the metal site in these solid solutions allows for tuning of bulk magnetic properties, including magnetic ordering temperature, transition temperatures and the nature of nuclear phase transitions, through adjustment of metal ratios.

## Abstract 2

We show that the modulated phase transitions in solid solutions of [CH_3_NH_3_]Co_*x*_Ni_1−*x*_(HCOO)_3_, with *x* = 0.25 (**1**), *x* = 0.50 (**2**) and *x* = 0.75 (**3**), can be tuned by the metal ratio, which offers the opportunity to consciously build molecular compounds with adjustable properties by doping metal sites.

## 1. Introduction

Coordination polymers (CPs) can integrate multiple physical properties into a single framework (Cui *et al.*, 2016 ▸; Lin *et al.*, 2014 ▸; Li *et al.*, 2016 ▸; Liu *et al.*, 2016 ▸; Furukawa *et al.*, 2013 ▸; Zhao & Miao, 2024 ▸; Wang *et al.*, 2024 ▸; Gomez-Romero *et al.*, 2024 ▸; Wang & Astruc, 2020 ▸). The magnetic properties of CPs are of particular interest, and can be combined with other physical characteristics to produce multifunctional materials. This multifunctionality is enabled by the presence of both organic molecules and metal cations in the same network and creates a plethora of opportunities for the development of novel smart materials (Coronado & Mínguez Espallargas, 2013 ▸; Luo *et al.*, 2016 ▸; Coronado, 2020 ▸; Verdaguer & Gleizes, 2020 ▸; Liu *et al.*, 2022 ▸). Aperiodic CPs are of growing interest to the crystallographic community as, despite being long-range ordered, they lack the three-dimensional periodicity which underlies many of the fundamental assumptions of diffraction analysis. Modulated crystals are an important class of aperiodic crystals (van Smaalen, 2004 ▸). A structure is modulated where the average translational symmetry is disrupted by the introduction of an additional periodic function. The modulation can describe the atomic displacements or occupations for structural modulations (Pinheiro & Abakumov, 2015 ▸; Janssen & Janner, 2014 ▸), where the periodicity of the modulations exceeds that of the average structure, *i.e.* the recurrent part of the structure is larger than the unit cell of the parent structure. If the modulation periodicity can be described by a rational fraction, the structure is commensurately modulated. If an irrational value is necessary, the compound is incommensurately modulated (van Smaalen, 2004 ▸).

The signature of modulated phases are satellite reflections in their diffraction patterns: reflections that cannot be indexed by a three-dimensional space group and are separate from the main Bragg reflection by a defined spacing. From the satellite reflections, the modulation periodicity, described by the wavevector **q**, can be calculated. The driving force behind the modulated phases lies in unresolved frustration (Dzyabchenko & Scheraga, 2004 ▸; Herbstein, 2005 ▸; Schönleber, 2011 ▸). Explored examples of mechanisms which have induced modulation include cooperative Jahn–Teller distortions (Noda *et al.*, 1978 ▸), inter/intra-molecular steric constraints (Bakus *et al.*, 2013 ▸) and hydrogen bonding (Canadillas-Delgado *et al.*, 2019 ▸). In each case, the competing interactions result in the loss of translational symmetry between average unit cells.

Reports of molecular frameworks that exhibit modulated phases are still limited (Aroyo *et al.*, 2011 ▸, 2006 ▸). This is particularly remarkable since weak interactions commonly observed in CPs, including hydrogen bonding, dipole–dipole interactions and π-stacking are equivalent to the forces that usually generate aperiodic systems (Pinheiro & Abakumov, 2015 ▸). This would suggest that many of the published compounds might have unreported modulated phases (Oppenheim *et al.*, 2020 ▸). However, the study of these systems is of potential interest because the intrinsic properties of the material, such as phonon, electric, magnetic, photonic or molecular transport properties, are likely to be different from those of periodic materials (Janssen & Janner, 2014 ▸; Poddubny & Ivchenko, 2010 ▸; de Regt *et al.*, 1995 ▸; Vardeny *et al.*, 2013 ▸). This is important in the sense that in many cases a more comprehensive review of the literature is necessary to identify novel structure–property relationships (Allendorf *et al.*, 2021 ▸).

**Figure 1:**
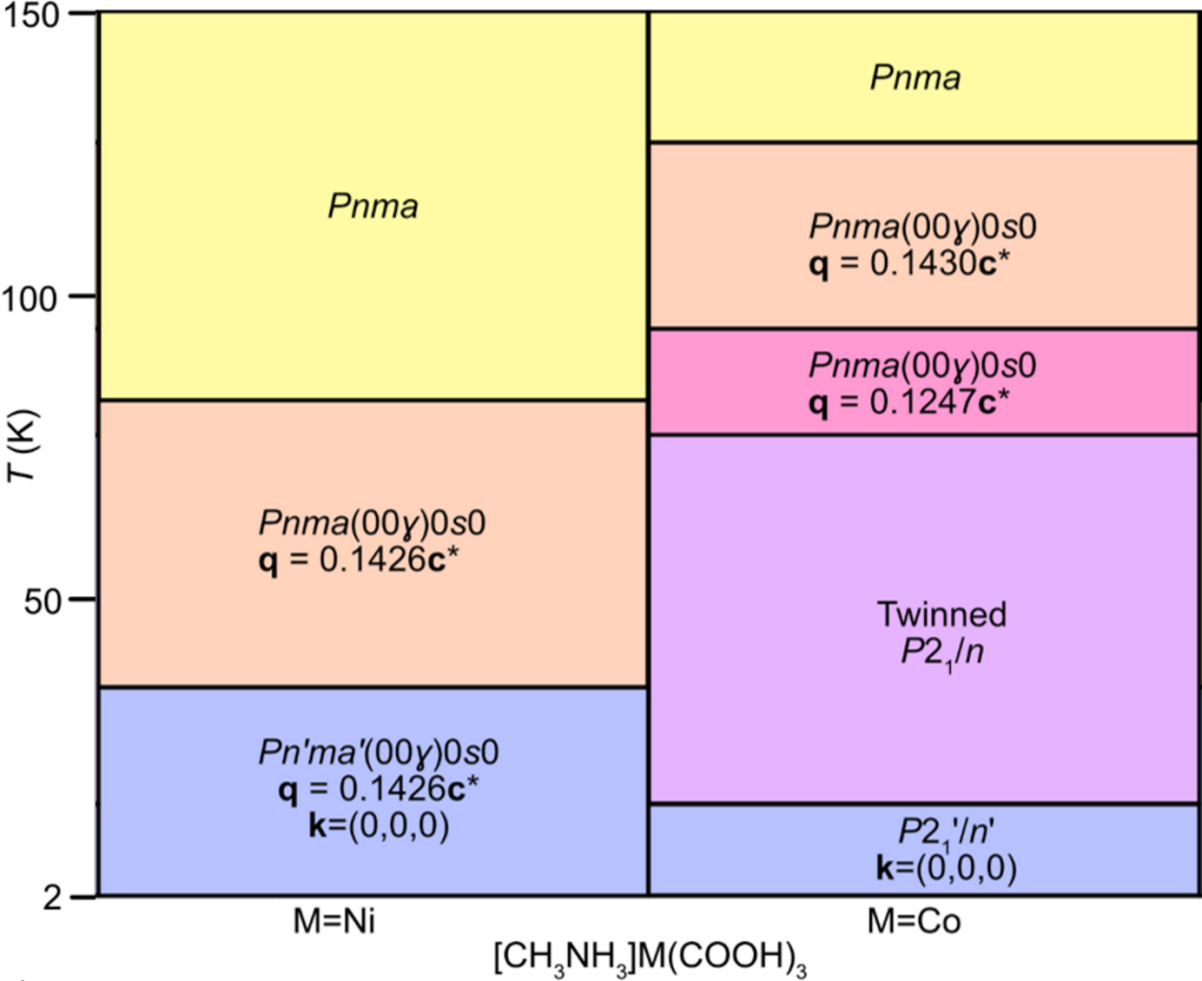
Temperature-dependent phase evolution for a single crystal of [CH_3_NH_3_]­Co(HCOO)_3_ and [CH_3_NH_3_]Ni(HCOO)_3_ from 2 to 150 K. Above 150 K up to ambient temperature, there are no further phase transitions observed. The lowest-temperature transitions for each metal (blue) correspond to magnetic ordering.

The methyl­ammonium metal formates, [CH_3_NH_3_]­*M*(HCOO)_3_, where *M* = Co and Ni (Canadillas-Delgado *et al.*, 2019 ▸, 2020 ▸), are more unusual, as both undergo phase transitions from unmodulated to incommensurately modulated structures on cooling. Although isomorphous at ambient temperature, the series of temperature-induced phase transitions exhibited by the two compounds are not equivalent (Fig. 1 ▸).

At ambient temperature [CH_3_NH_3_]*M*(HCOO)_3_ (*M* = Co and Ni) crystallizes in the orthorhombic space group *Pnma* (Boča *et al.*, 2004 ▸), adopting a perovskite-like structure *ABX*_3_, with the metal atoms occupying the *B*-site, the methyl­ammonium cation located in the *A*-site and the formate ligand serving as a linker between the metal atoms at the *X*-site. Each metal site (Wyckoff site 4*b*) is octahedrally coordinated and is bridged by formate anions in the anti–anti coordination mode to form a three-dimensional framework. The carbon and nitro­gen atoms of the methyl­ammonium cations are positioned at (*x*, 0.25, *z*) and (*x*, 0.75, *z*), within the voids of the framework. On cooling, [CH_3_NH_3_]Co(HCOO)_3_ experiences its first phase transition at approximately 128 K to the superspace group *Pnma*(00γ)0*s*0 (Canadillas-Delgado *et al.*, 2019 ▸). In this phase, there is a modulated unit cell, with the wavevector **q** = 0.1430 (2)c*, describing a modulation length of 6.99 times that of the average *c* axis. At 96 K, a second transition is observed where there is a change in the wavevector **q** = 0.1247 (2)c*, however the symmetry of the crystal remains the same, *Pnma*(00γ)0*s*0. In this phase, the modulation length is approximately 7.92 times larger than the average unit cell. For both the modulated phases, the atom displacement occurs predominately along the *b* axis, with the amplitude displacements larger for the second modulated phase. Below 78 K a fourth phase is obtained, a twinned, non-modulated monoclinic structure with *P*2_1_/*n* symmetry (Mazzuca *et al.*, 2018 ▸), with two domains, contributing 50% to the total intensity, related by a rotation of 180° around the orthorhombic c* axis. The onset of long-range magnetic ordering with weak ferromagnetic behaviour is observed at 16 K, with the ferromagnetic component along the *c* axis (Gómez-Aguirre *et al.*, 2016 ▸; Pato-Doldán *et al.*, 2016 ▸; Ding *et al.*, 2023 ▸). In the single-crystal study, only the monoclinic *P*2_1_′/*n*′ magnetic structure is observed (Canadillas-Delgado *et al.*, 2019 ▸) [Fig. S1(*a*) of the supporting information]; however, with powder neutron diffraction data a combination of *P*2_1_′/*n*′ and *Pn′ma′* magnetic phases is reported (Mazzuca *et al.*, 2018 ▸).

In comparison, the Ni analogue remains in the non-modulated *Pnma* space group on cooling to 84 K (Cañadillas-Delgado *et al.*, 2020 ▸). Below this temperature, it adopts the superspace group *Pnma*(00γ)0*s*0 with **q** = 0.1426 (2)c*. [CH_3_NH_3_]Ni(HCOO)_3_ remains in this incommensurately modulated phase until the onset of long-range magnetic ordering at 34 K (Pato-Doldán *et al.*, 2016 ▸), where the compound orders in the magnetic superspace group *Pn′ma′*(00γ)0*s*0. Here, the phase is described as a proper incommensurate magnetic structure, as the magnetic moments network presents a modulation due to the occurrence of incommensurate magnetic modes. Consequently, in this phase there is the coexistence of an incommensurately modulated nuclear and magnetic structure. The moments are orientated primarily along the *c* axis, with an uncompensated contribution in the *b* direction [Figs. S1(*b*) and S1(*c*)]. The modulation of the moments occurs as static librations in the *ac* plane. The stimulus driving the modulated phase transitions for both the formate compounds is the hydrogen-bonded network between the NH_3_ hydrogen atoms of the methyl­ammonium cations and the oxygen atoms of the formate ligands. In the ambient-temperature phase, two of the hydrogen atoms participate in hydrogen bond interactions, whilst the third is too far in proximity to interact with the neighbouring oxygen atoms [Fig. S2(*a*)]. In the low-temperature *P*2_1_/*n* phase, adopted by [CH_3_NH_3_]Co(HCOO)_3_, a third permanent hydrogen bond is present [Fig. S2(*b*)]. The methyl­ammonium cation, which is positioned along a mirror plane in the orthorhombic phase, rotates in the monoclinic phase breaking the symmetry element. H1 [atom labels as in Fig. S2(*d*)], which was originally equidistant to O3 and O3a (with *a* = *x*, −*y* + 3/2, *z*), is now closer to one of the oxygen atoms, establishing a hydrogen bond interaction. In the modulated phases, H1 is close enough in distance to interact with the oxygen atoms of the formate ligand. However, there are two competing hydrogen-bond acceptors [O3 and O3a, Fig. S2(*c*)] that are equidistant from H1 in the average structure. This frustration distorts the structure and results in the modulated displacement of all the atoms. Consequently, at a given point in the structure, O3 will be closer in distance to H1, yet, in other areas along the modulation, the N1—H1⋯O3a distance will be shorter.

In recent work on the control of the modulated phases (Li *et al.*, 2020 ▸), the authors analyse a metal–organic framework material, MFM-520, which displays a reversible periodic-to-aperiodic structural transition through host–guest interaction. The dehydrated phase presents an aperiodic structure with translational symmetry in (3+2)D space, which changes to a periodic phase when H_2_O molecules are incorporated in the pores of the structure. Subsequent substitution of H_2_O molecules with CO_2_ and SO_2_ revealed that, while CO_2_ exerts minimal structural influence, SO_2_ can also induce modulation in the structure. This study motivated us to investigate the feasibility of combining Ni and Co in the perovskite *B*-site to explore the sensitivity of the modulated phase transitions and magnetic characteristics of the formate compounds.

In the present work, solid solutions have been synthesized from the methyl­ammonium metal formates, [CH_3_NH_3_]­Co*_x_*Ni_1−*x*_(HCOO)_3_, with *x* = 0.25 (**1**), *x* = 0.50 (**2**) and *x* = 0.75 (**3**), and their structures and magnetic properties have been analysed through single-crystal neutron diffraction and magnetometry studies. All three compounds were studied through Laue experiments on CYCLOPS and monochromatic measurements on D9 instruments, and a deep study of the modulated phases and magnetic structure was carried out on compound **2** on the D19 monochromatic diffractometer. Magnetic measurements were carried out using a Quantum Design Magnetic Property Measurements System-XL (MPMS) with a Superconducting Quantum Interference Device (SQUID) magnetometer.

## 2. Results

### 2.1. Synthesis and ambient structure

Methyl­ammonium formate solid solutions were synthesized under solvothermal conditions from aqueous solutions of NiCl_2_·6H_2_O, CoCl_2_·6H_2_O, CH_3_NH_3_Cl and NaHCOO in stoichiometric quantities following the method previously reported (Mazzuca *et al.*, 2018 ▸; Wang *et al.*, 2004 ▸). The solutions were heated at 413 K (140°C) for 72 h before cooling, within the sealed autoclave, to room temperature. This yielded mm^3^-sized crystals of **1** (dark green), **2** (dark green) and **3** (dark red).

**Figure 2:**
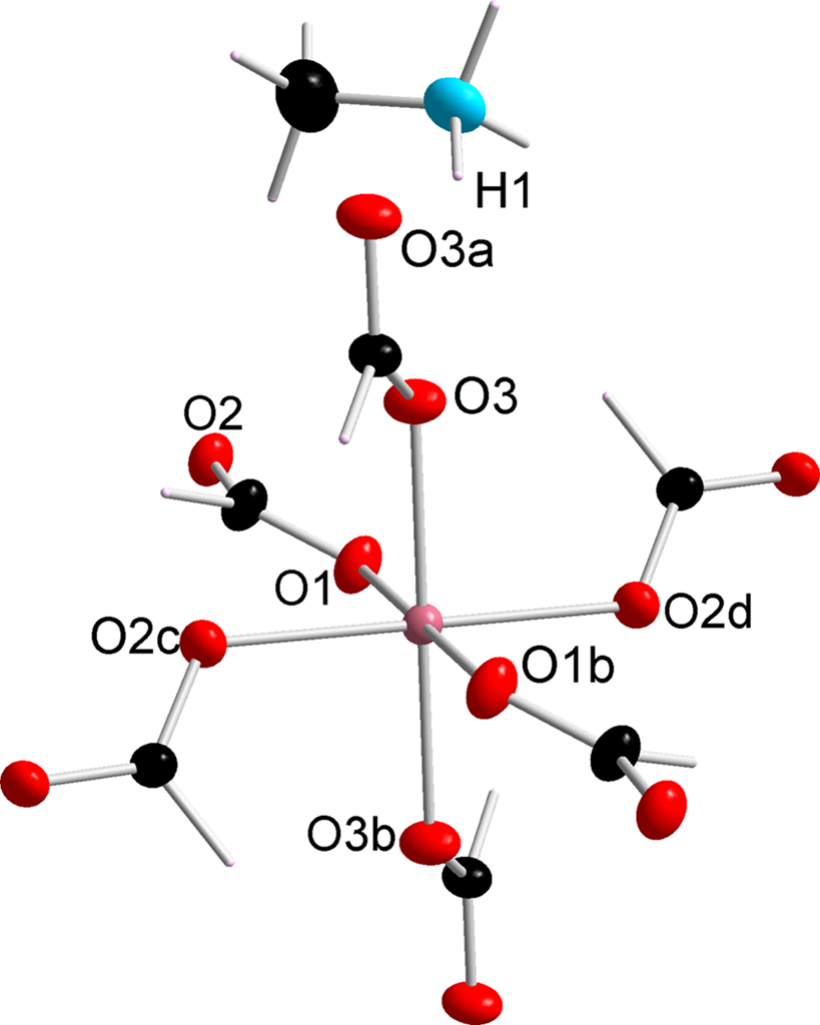
Connectivity of the metal–formate framework for compound **2** measured with the D9 diffractometer (ILL) at ambient temperature. Compounds **1** and **3** are isostructural. The ellipsoids are drawn with 50% probability and the hydrogen atoms are represented as sticks for clarity. Colour code: metal = pink, O – red, C – black, N – blue. Symmetry code: *a* = *x*, −*y* + 3/2, *z*; *b* = −*x* + 1, −*y* + 1, −*z* + 2; *c* = −*x* + 1/2, −*y* + 1, *z* + 1/2; *d* = *x* + 1/2, *y*, −*z* + 3/2.

Single-crystal X-ray diffraction measurements reveal that the compounds are isostructural to the end-member methyl­ammonium formate compounds [CH_3_NH_3_]Co(HCOO)_3_ and [CH_3_NH_3_]Ni(HCOO)_3_ (Boča *et al.*, 2004 ▸; Pato-Doldán *et al.*, 2016 ▸) (Fig. 2 ▸). Neutron diffraction studies were performed using the D9 diffractometer at the ILL to obtain more precise information on the metal site orderings and the Co and Ni ratio in each crystal (Geers & Cañadillas-Delgado, 2021*b* ▸; Cañadillas-Delgado *et al.*, 2023 ▸). Room-temperature diffraction data were collected for single crystals of **1** (2 × 1.5 × 1.5 mm^3^), **2** (3 × 2 × 2 mm^3^) and **3** (1.5 × 1.5 × 1 mm^3^). The Co and Ni occupations were refined and were constrained such that the overall site occupancy was 1 and while the site positions and anisotropic displacement parameters for both species were constrained to be equal.

The neutron diffraction data show that there is no ordering of the metals. The symmetry for the solid solutions are the same as for the end members, *Pnma*, with no superlattice reflections observed or reflections corresponding to systematic absences in the *Pnma* space group. The metal content for each crystal corresponds to *x* = 0.297 (9), 0.526 (8) and 0.765 (20) for **1**, **2** and **3**, respectively. These values are close to the stoichiometric quantities of metal chlorides used in the synthesis.

### 2.2. Magnetometry

Field-cooled (FC) and zero-field-cooled (ZFC) susceptibility measurements were carried out on microcrystalline samples using an applied field of 100 Oe over the temperature range 2–300 K. The extent of field induced magnetization was also explored at 2 K between −5.00 (1) and 5.00 (1) T.

**Figure 3:**
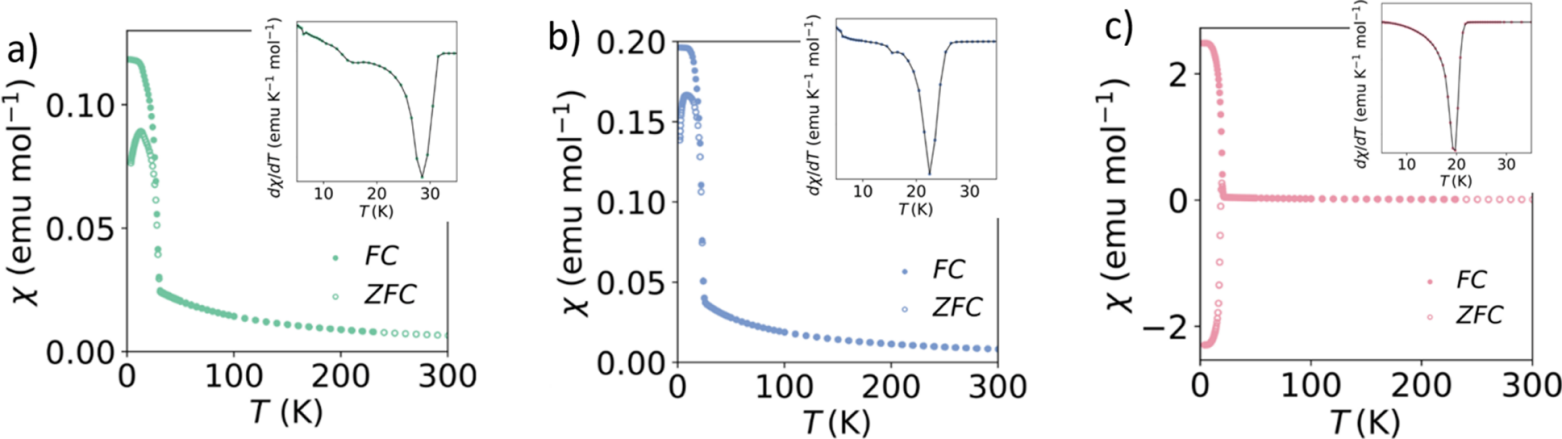
FC and ZFC susceptibilities for compounds (*a*) **1**, (*b*) **2** and (*c*) **3**. The insets correspond to the derivative of the susceptibility.

The susceptibility data for compound **1** (Co_25_Ni_75_) indicate an ordering temperature *T*_C_ = 28.5 (5) K [Fig. 3 ▸(*a*)]. From the plot of χ*T*, the effective moment is calculated to be 3.936 (2) μ_B_ in comparison with μ_spin only_ = 3.09 μ_B_ and the Curie–Weiss temperature θ_CW_ = −70.8 (7) K (150 < *T* < 300 K). The high-temperature Curie constant, *C*, extracted from the Curie–Weiss fit in the range from 150 to 300 K is 2.432 (7) emu K mol^−1^ [Fig. S3(*a*)] which is larger than the spin-only value *C*_spin only_ = 1.21 emu K mol^−1^. This discrepancy in the Curie constant with respect to the spin-only value indicates strong orbital contributions, as is also reflected in the pure Co compound (Pato-Doldán *et al.*, 2016 ▸), and is also present in the other solid solutions (**2** and **3**). Both Ni^2+^ and Co^2+^ ions have large orbital contributions to the magnetic moment, meaning that in all these formate compounds θ_CW_ varied greatly with the temperature range used to perform the fit. The isothermal data reveal hysteresis at all magnetic fields measured for **1** [Fig. S4(*a*)]. It exhibits a remnant magnetization *M*_rem_ = 0.015 (1) μ_B_ per metal and a coercive field of *H*_C_ = 0.15 (1) T. The magnetization does not reach saturation, *M*_sat_ = 1.125 (1) μ_B_ per metal, with *M*_5T_ = 0.158 (1) μ_B_ per metal at 5.00 (1) T (*M*_5T_/*M*_sat._ = 0.140).

For compound **2** (Co_50_Ni_50_), the FC and ZFC susceptibilities diverge at the ordering temperature, *T*_C_ = 22.5 (7) K [Fig. 3 ▸(*b*)]. The effective magnetic moment μ_eff_ = 4.436 (2) μ_B_ compared with μ_spin only_ = 3.35 μ_B_. Fitting the inverse susceptibility to the Curie–Weiss Law we obtain θ_CW_ = −56.3 (1) K and *C* = 2.938 (1) emu K mol^−1^ in the range 150 < *T* < 300 K, which is significantly higher than the spin only value *C*_spin only_ = 1.44 emu K mol^−1^ [Fig. S3(*b*)]. Hysteresis is observed in the magnetization data of **2**, which closes at 5.0 (2) T [Fig. S4(*b*)]. The remnant magnetization is *M*_rem_ = 0.027 (1) μ_B_ per metal, with a coercive field of *H*_C_ = 0.30 (1) T. The largest magnetization measured is *M*_5T_ = 0.218 (1) μ_B_ per metal, which is lower than the saturation value *M*_sat_ = 1.25 μ_B_ per metal (*M*_5T_/*M*_sat_ = 0.175).

As compound **3** (Co_75_Ni_25_) is cooled, the FC and ZFC susceptibilities split at the ordering temperature, *T*_C_ = 19.7 (5) K [Fig. 3 ▸(*c*)]. Similarly, the effective moment, μ_eff_ = 4.578 (2) μ_B_, is larger than the spin-only value 3.61 μ_B_. The Curie–Weiss temperature, obtained from a high-temperature fit of the data 150 < *T* < 300 K, is θ_CW_ = −43.6 (5) K, and *C* = 2.997 (3) emu K mol^−1^ in the same temperature range, which is greater than the spin-only value for the average magnetic site *C*_spin only_ = 1.66 emu K mol^−1^ [Fig. S3(*c*)]. The isothermal magnetization measurements for compound **3** show that as the field is increased, hysteresis is observed up to 3.04 (1) T, where the magnetization *M* = 0.219 (1) μ_B_ per metal [Fig. S4(*c*)]. The remnant magnetization is *M*_rem._ = 0.045 (3) μ_B_ per metal, and the hysteresis has a coercive field of *H*_C_ = 0.45 (1) T. At the largest field measured, 5.00 (1) T, the magnetization reaches *M*_5 T_ = 0.337 (2) μ_B_ per metal, with no signs of a plateau. The degree of saturation is *M*_5T_/*M*_sat_ = 0.245, where the saturation value *M*_sat_ = 1.375 μ_B_ per metal.

For all three compounds, the combination of a negative θ_CW_ and hysteresis in the isothermal data suggest weak ferromagnetic behaviour, in agreement with the magnetic ordering of the parent compounds. If the crystals were biphasic, rather than true solid solutions, it would be expected that there are two ordering temperatures observed in the magnetometry data, one for the pure Co compound at 16 K and one for the pure Ni compound at 34 K. For the samples measured, essentially one ordering temperature is observed which increases incrementally between the Co and Ni ordering temperatures, dependent on the metal ratios used in the synthesis. This would support the X-ray and neutron diffraction data that there is no metal site ordering and the distribution of Co and Ni is random within the samples.

### 2.3. Temperature-dependent structural evolutions

Single-crystal Laue neutron diffraction was carried out for compounds **1**, **2** and **3** on the CYCLOPS diffractometer at ILL, with the wavelength range 0.8−3.0 Å on the same crystals used on D9 diffractometer (Geers & Cañadillas-Delgado, 2021*a* ▸). The samples were heated between 10 and 120 K and the diffractograms were collected with a 3 K range per image. This technique was not used to determine the structures, but to determine the temperatures at which structural phase transitions appear and estimate the **q** vectors of modulated phases.

For the Ni-rich compound, **1**, the crystal remains in the *Pnma* space group on cooling to 85 (3) K. At this temperature, satellite reflections are visible (Fig. S5). The reflections match a calculated diffraction pattern for the *Pnma* space group with **q** = 0.140 (5)c*. These reflections remain as the sample is cooled to 10 K, without observable alteration to the **q** vector. Below 28 (3) K, although no new reflections appear in the pattern of the selected orientation, certain reflections increase in intensity [Fig. S5(*e*)]. This observation aligns with the long-range magnetic ordering temperature identified through magnetometry measurements (*T*_C_ = 28.5 (5) K), indicating a **k** = (0,0,0) propagation vector.

When cooling, compound **2** remains in a non-modulated *Pnma* phase to 96 (3) K, at which temperature satellite reflections appear and the main reflection reduces in intensity [Fig. S6(*b*)]. The reflections can be modelled by the *Pnma* space group, with **q** = 0.140 (5)c*. A smooth phase transition can be observed at 59 (3) K with a change in distance between the main and satellite reflections as well as the appearance of additional satellite reflections, which increase in intensity down to 33 (3) K [Fig. S6(*c*)]. The reflections can be matched to the *Pnma* space group with **q** = 0.120 (5)c*. Below this temperature the position of the main and satellite reflections do not change further. Additional intensity to some reflections can be observed below 25 (3) K, in line with the ordering temperature observed from the magnetization data [*T*_C_ = 22.5 (7) K, Fig. S6(*d*)]. This implies that the magnetic structure has a propagation vector of **k** = (0,0,0).

Cooling from 120 K, the diffraction pattern for **3** remains unchanged in the non-modulated *Pnma* space group to 98 (3) K, where the emergence of satellite peaks can be observed [Fig. S7(*b*)]. Below 98 (3) K, the main Bragg reflections match a calculated diffraction pattern for the *Pnma* space group and the satellite peaks agree with a **q** vector of about 0.135 (5)c*, which subtly decreases to **q** = 0.125 (5)c* by 58 (3) K. From 58 (3) K a slow transition occurs over a 14 (3) K temperature range [Fig. S7(*c*)]. During the transition, the reflections reduce in intensity and are distributed over a larger pixel area. By 44 (3) K, the reflections are again sharp intensities. The reflections no longer have satellite peaks, suggesting this is a non-modulated structure, and twinning can be observed by the appearance of a second Bragg reflection close in proximity to the main reflection [Fig. S7(*d*)]. The reflections fit the monoclinic space group *P*2_1_/*n*, like the low-temperature phase of the pure cobalt formate compound. At 18 (3) K, certain reflections increase in intensity, indicating the onset of long-range magnetic ordering, which is in agreement with the ordering temperature determined by the magnetometry measurements (*T*_C_ = 19.7 (5) K). Since the magnetic reflections only appear as additional intensity to nuclear reflections, it likely has a propagation vector of **k** = (0,0,0).

### 2.4. Monochromatic neutron diffraction

The Laue diffraction data show that the Co-rich and Ni-rich solid solutions follow structural phase transitions similar to that of the end-member methyl­ammonium formates. However, the evolution of the Co_50_Ni_50_ (compound **2**) structure did not strictly follow the trend for either the Co or the Ni analogue, encouraging a further neutron diffraction experiment to explain the observed behaviour.

A single-crystal neutron diffraction experiment was carried out using the D19 diffractometer (ILL), using the same crystal that was used on the CYCLOPS and D9 diffractometers (Geers *et al.*, 2021 ▸). Data were collected at 2 and 10 K intervals between 30 and 100 K. The orientation matrix was obtained for each dataset to identify the temperature regimes for each phase. As a result, longer data acquisitions were made at 30 and 70 K. Refinements were carried out at 70, 30 and 2 K to determine the nuclear and magnetic structures of each phase. For the following refinements, the previously calculated metal ratio for this crystal was used (0.526:0.474, Co:Ni) and were fixed during the refinements. A summary of the experimental and crystallographic data can be consulted in Table 1 ▸.

At 96 (3) K, compound **2** undergoes a phase transition from the non-modulated, orthorhombic space group *Pnma* to a modulated structure. Integration of the data at 70 K found that the compound adopts the modulated superspace group *Pnma*(00γ)0*s*0 with a modulation vector **q** = 0.1429 (2)c*.

Refinements of the amplitude displacements for the atoms at 70 K reveal that the site displacement is largest along the *b* direction. The modulation for each atom was refined independently to find the displacements. For the metal site, the displacement is described by a sine term only (restricted by symmetry, Table 2 ▸), with a maximum displacement from its average position of 0.235 (3) Å in the *b* direction (Fig. S8). Asynchronous modulations of the atoms result in the modulation of the bond lengths; however, these variations are of two orders of magnitude smaller than the displacements experienced for the atoms.

The hydrogen bond interactions between the donor N—H methyl­ammonium and acceptor O atoms vary in distance as the structure modulates. The H2⋯O2 interactions [atoms labelled as in Fig. S2(*d*)] vary in the range from 1.807 (3) to 1.830 (3) Å, indicating that both atoms preserve an appropriate distance to maintain hydrogen bonding in all regions of the crystal. The H1⋯O3 and H1⋯O3a distances vary between 2.028 (3) and 2.265 (3) Å. This variation of distances gives rise to flip-flop behaviour, so that in some regions of the crystal a possible hydrogen bond is established with O3, in other regions a minimum distance is established with O3a and in other regions the distances to O3 and O3a are sufficiently long to dismiss the hydrogen bond.

Decreasing in temperature, a second phase transition is observed between 40 and 30 K. This is slightly lower than the transition observed in the Laue diffraction data at 59 (3) K. This discrepancy in the transition temperature is potentially the result of the temperature continually increasing as a function of time for the Laue diffraction measurements, whereas during these data collections the sample was allocated time to stabilize at each temperature point before starting the data collection.

At 30 K, compound **2** presents a crystal structure refined in the superspace group *Pnma*(00γ)0*s*0, with a modulation vector **q** = 0.1249 (2)c*. Although new satellite reflections appeared in the Laue measurement below 59 (3) K, monochromatic measurements revealed that all the satellite reflections in this phase are first and second order, like in the previous measurement at 70 K. This contrasts with the measurement of the pure Co compound where third-order satellites were visible below 90 K, *i.e.* the temperature corresponding to the change of the wavevector from **q** = 0.1430 (2)c* to **q** = 0.1247 (2)c*. The lack of third-order satellites is likely due to compound **2** being a solid solution which could tend to reduces homogeneity and the overall crystallinity of the sample. Like in the previous described phase, the metal site positions modulate with the largest contribution along the *b* direction (Table 2 ▸). This results in a maximum displacement along the *b* axis from its average position of 0.373 (3) Å (Fig. S8).

The *M*—O bond lengths exhibit larger distortions from the average value at 30 K compared with the 70 K phase. This is particularly significant for *M*—O1 and *M*—O3 (Table S2 of the supporting information). *M*—O3 coordinate to the metal sites along the *b* direction, with a maximum deviation from the average bond length of 0.069 (5) Å. The *M*—O1 bonds are located in the *ab* plane with a maximum bond length variation of 0.060 (3) Å. The difference in maximum bond length for *M*–O2 compared with its average value is an order of magnitude smaller, 0.007 (3) Å. The modulated distance H2⋯O2 ranges from 1.784 (4) to 1.863 (3) Å, indicating that the interaction is present throughout the crystal. The H1⋯O3 and H1⋯O3a modulations alternate, resulting in minimum and maximum distances at different points in the structure, as in the previous phase [Fig. S9(*b*)].

On further cooling to 2 K, additional reflections were observed, indicating the onset of magnetic ordering. The presence of the satellite reflections implies that the structure remains in a modulated phase. However, initially it was unclear if the modulation arises from only the nuclear structure (improper incommensurate magnetic structure) or from a combination of the nuclear and magnetic structure (proper incommensurate magnetic structure). Refinements were carried out both where the Fourier components for the magnetic moments were refined and where the modulations were fixed to zero for the moments. As equivalent refinement statistics were obtained for both models, and there was no evidence of additional intensities in the satellite reflections, but instead only an increase in intensities of the main reflections, it was concluded the structure adopts an improper modulated magnetic structure.

**Figure 4:**
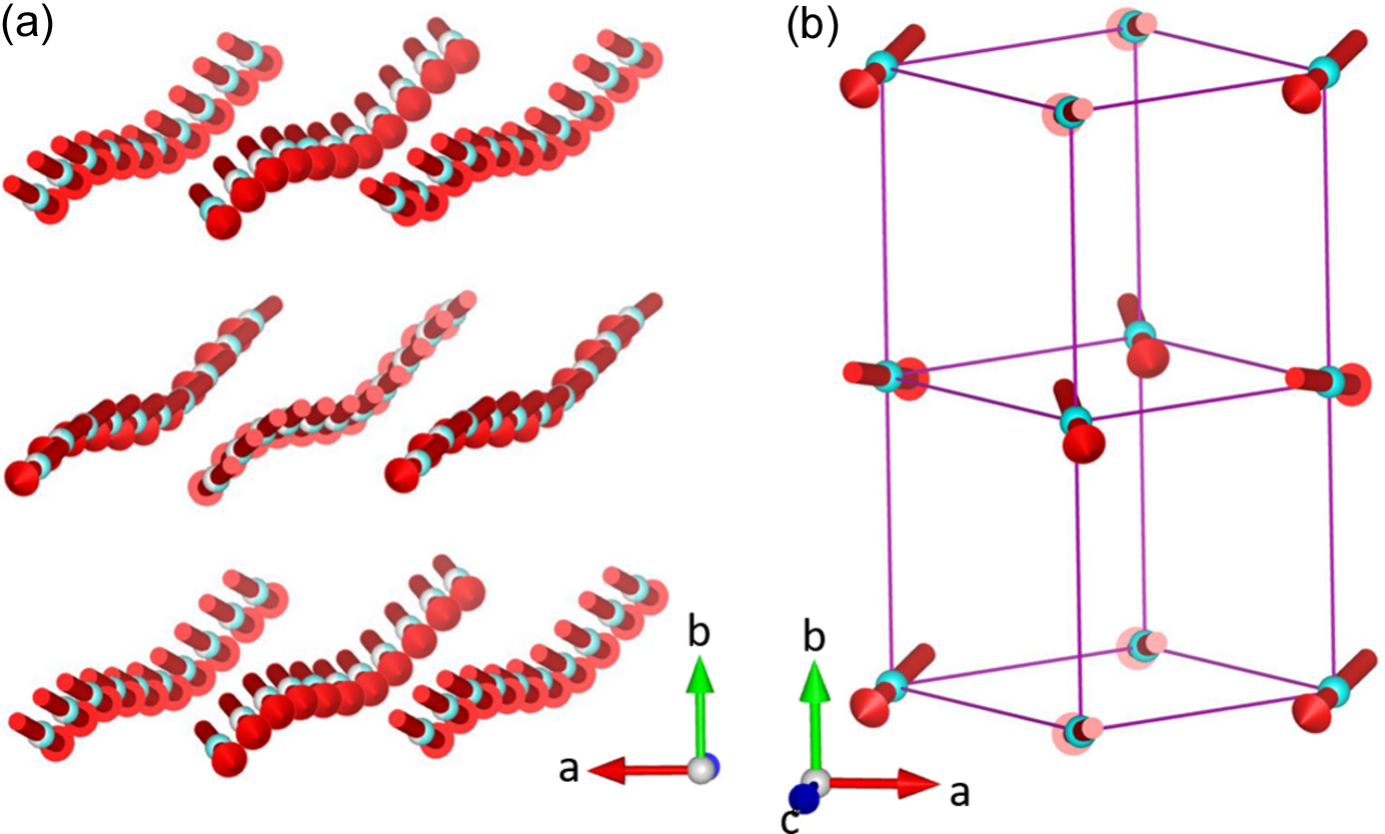
Magnetic structure of compound **2** at 2 K measured with the D19 diffractometer (ILL). (*a*) The position of the metal sites and the magnetic moments are represented in a supercell which is 10 times larger than the average unit cell to include at least a full modulation period. (*b*) Average magnetic unit cell, showing the ordering of the moments without the modulation of the atom sites. The [*M*(HCOO)_3_]^−^ framework is represented as a wireframe (pink lines) and the methyl­ammonium cations have been removed for clarity. The moments are tilted, with an uncompensated moment along the *b* axis.

From indexing the magnetic Bragg reflections, the propagation vector was determined to be **k** = (0,0,0). Compound **2** orders with the magnetic superspace group *Pn′ma′*(00γ)0*s*0 with **q** = 0.1249 (2)c*. This model allows for a weak ferromagnetic arrangement of the moment, in agreement with the susceptibility data. The moment was fixed to have a magnitude of 2.50 (2) μ_B_, an average of high-spin Co^2+^ and Ni^2+^ moments. Each nearest neighbour, through *M*—OCO—*M* superexchange pathways, has weak ferromagnetic correlations, caused by a canting of the moments along the *b* axis at an angle of *ca* 104° (0.6 (2) μ_B_) with respect to this axis (Fig. 4 ▸). Since the magnetic component of the structure is non-modulated, the orientation and size of the moment does not change throughout the structure. The nuclear component, however, remains modulated. The metal site modulates and, accordingly, the position of the moment is displaced from the average structure, however, the magnitude and direction of the moments are not varied. The greatest displacement of the atom sites is along the *b* axis (Table 2 ▸), with a maximum displacement of 0.388 (6) Å (Fig. S8, blue line).

The length of the *c* axis, as well as the unit-cell volume in the modulated phases, is 7 and 8 times larger than in the commensurate orthorhombic phase (*Pnma* phase). In these cases, a refinement in a non-modulated phase is possible, however the number of parameters becomes too large, so that the number of measured reflections is not sufficient and the refinement becomes unstable. This has already been tested in the pure Co compound (Canadillas-Delgado *et al.*, 2019 ▸), where it is observed that the space group would become *P*2_1_2_1_2_1_, with a number of atoms in the asymmetric unit equal to 147, compared with the 14 atoms of the asymmetric unit in the supergroup.

## 3. Discussion

**Figure 5:**
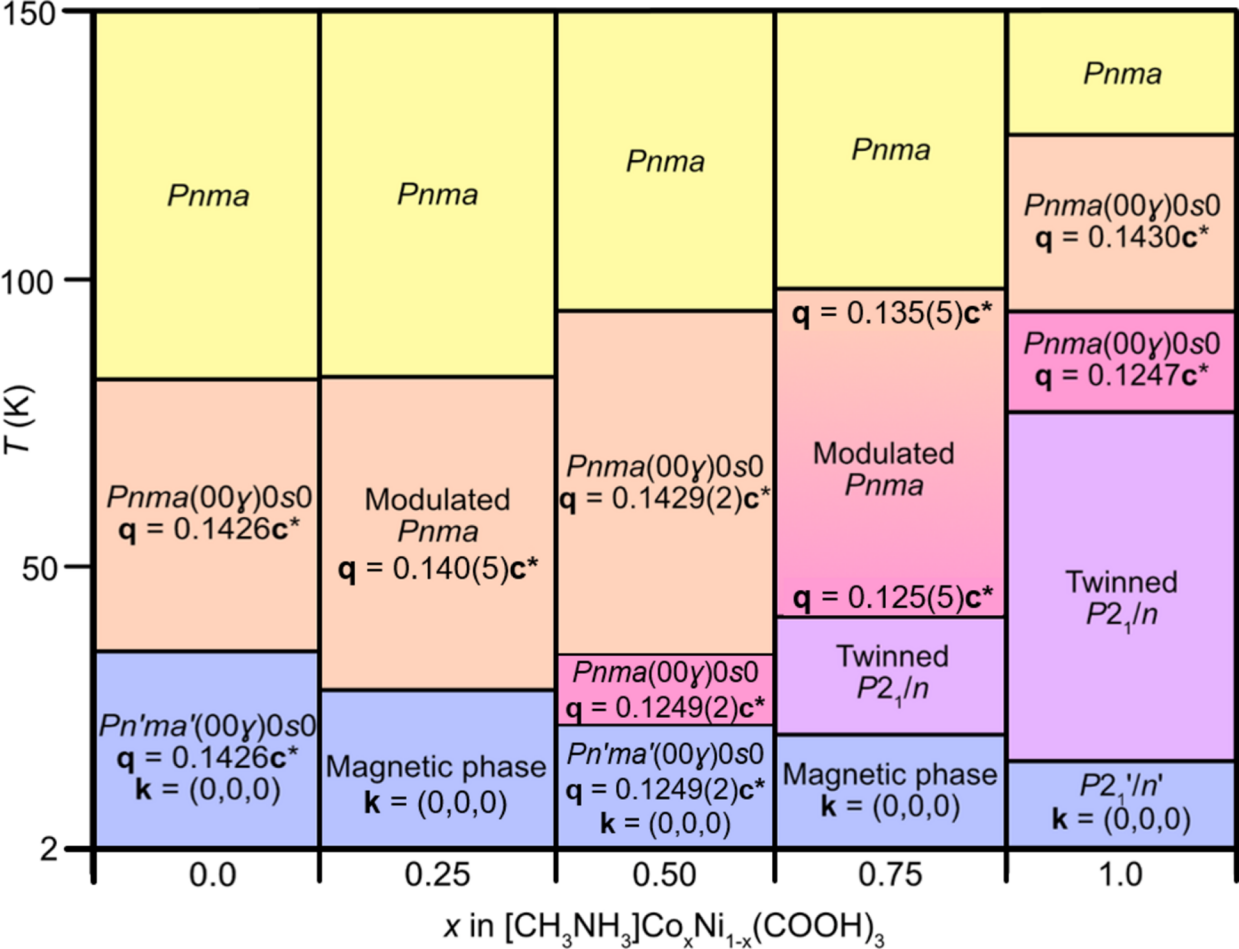
Summary of the structural and magnetic temperature-dependent phase transitions in single-crystal samples of [CH_3_NH_3_]Co*_x_*Ni_1−*x*_(HCOO)_3_ with *x* = 0, *x* = 0.25 (**1**), *x* = 0.5 (**2**), *x* = 0.75 (**3**) and *x* = 1. For compounds **1** and **3**, the transition temperatures were obtained from neutron Laue diffraction measurements, and from monochromatic neutron diffraction measurements for compound **2**. The temperature of the magnetic order (phases represented in blue) are obtained from the magnetometry data.

The three solid solutions of [CH_3_NH_3_]Co*_x_*Ni_1−*x*_(HCOO)_3_, *x* = 0.25 (**1**), *x* = 0.50 (**2**) and *x* = 0.75 (**3**), show intermediate behaviours of their structural evolutions and magnetic properties compared with their two end members [CH_3_NH_3_]­Co(HCOO)_3_ and [CH_3_NH_3_]Ni(HCOO)_3_. The Ni-rich compound **1** undergoes one non-modulated to modulated phase transition, which remains until 2 K. This behaviour follows the transitions observed by the Ni analogue. On the other hand, compound **3** exhibits structural phase transitions similar to that of the Co analogue, transitioning through modulated phases before adopting a twinned non-modulated structure by 44 (3) K (Fig. 5 ▸). The wavevector of the modulated phase just below 98 (3) K, **q** = 0.135 (5)c* corresponds to an incommensurate modulated structure, rather than near commensurate, as in the rest of compounds. It presents smooth phase transitions in wide ranges of temperatures, together with a distribution of the reflections over a large pixel area in the Laue measurements that would imply the presence of several domains in the sample. The coexistence of phases appears recurrently in all compounds of this family suggesting that the small energy barrier between phases could be easily overcome by external stimuli such as external pressure. Recently, the effect of external pressure on the parent compound [CH_3_NH_3_]Co(HCOO)_3_ has been studied using high-pressure powder X-ray diffraction and Raman spectroscopy (Zhou *et al.*, 2023 ▸). The increase in pressure at room temperature gives rise to a phase transition from the ortho­rhombic *Pnma* to a monoclinic phase, at approximately 6.13 GPa. This study indicates that high pressure can profoundly alter the crystal structure and magnetic properties of these compounds, implying that this external stimulus can also serve to control also the phase transition from the modulate structure at low temperature.

The structural behaviour of **2** exhibits similarities to both the Co and the Ni parent compounds, yet the series of phase transitions do not follow either compound directly. The first two phase transitions, between the non-modulated phase and a modulated phase with **q** = 0.1429 (2)c*, followed by an isomorphous phase transition to a structure with **q** = 0.1249 (2)c*, resemble that of the Co analogue, although occurring at lower temperatures. Compared with the Co analogue, which undergoes a transition to a twinned non-modulated monoclinic structure that is retained with the onset of magnetic ordering, compound **2** does not exhibit a low-temperature non-modulated phase. It magnetically orders in the superspace group *Pn*′*ma*′(00γ)0*s*0 with **q** = 0.1249 (2)c* and **k** = (0,0,0). The magnetic symmetry is similar to that of the Ni analogue, although with a smaller modulation vector and only the nuclear structure that contributes to the modulations, the magnetic ordering is non-modulated.

Note that Laue measurements reveal a significant increase in the temperature range at which modulated structures manifest in solid solution compounds, in contrast to pure Ni and Co compounds. Specifically, although the temperature range spans 82 and 50 K for pure Ni and Co compounds, respectively, compounds **1**, **2** and **3** exhibit an extended temperature range reaching approximately 83, 94 and 54 K, respectively. This suggests that doping the samples increases frustration in the structure, leading to the stabilization of modulated structures.

**Figure 6:**
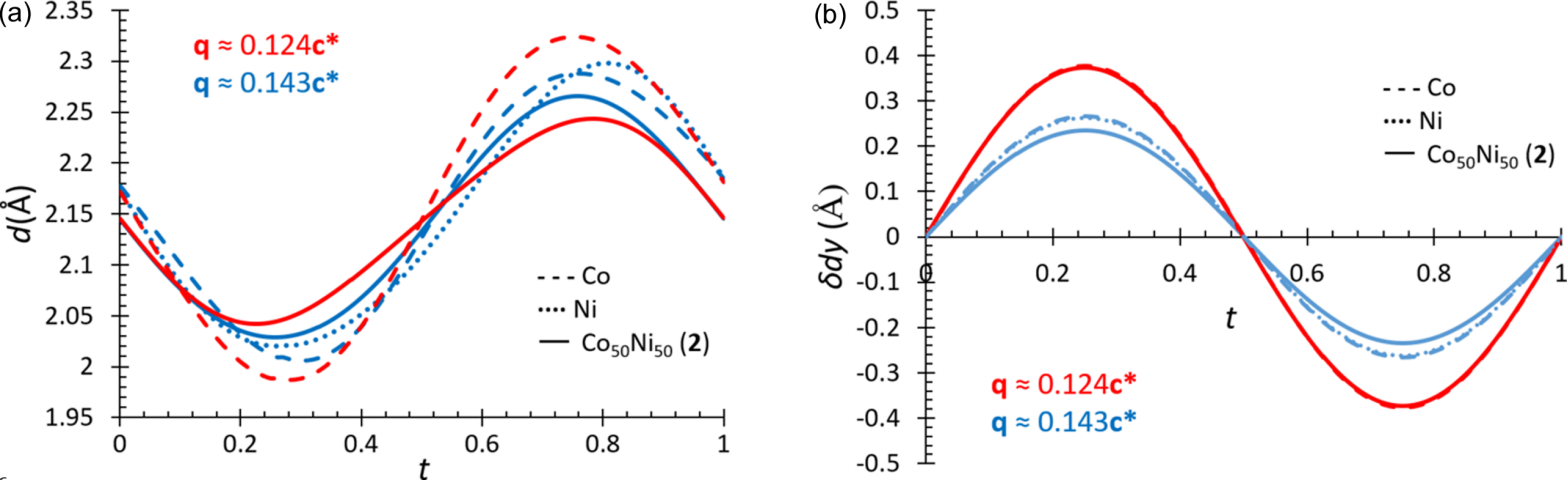
Summary of the (*a*) H1⋯O3 bond distances and (*b*) metal displacement along *b* axis for [CH_3_NH_3_]Co(HCOO)_3_ at 106 (blue) and 86 K (red) (dash lines) (Canadillas-Delgado *et al.*, 2019 ▸), [CH_3_NH_3_]Ni(HCOO)_3_ at 40 K (blue dotted lines) (Cañadillas-Delgado *et al.*, 2020 ▸) and compound **2** at 70 (blue) and 30 K (red) (solid lines). Red lines highlight the structures with the approximate wavevector **q** = 0.124c* and the blue lines for the approximate wavevector **q** = 0.143c*.

From the low-temperature monochromatic neutron diffraction data, it can be extracted that the mechanism inducing the modulated phase transitions in **2** is the competition of the hydrogen bonding interactions, akin to its parent compounds. Although hydrogen bonding might not be essential in halide perovskites, research demonstrates that it is approximately three times more robust in formate perovskites (Svane *et al.*, 2017 ▸). The H2⋯O2 distance remains at values close to its average value, with only small deviations of up to ±0.040 (4) Å. H1⋯O3, which denotes the hydrogen bonding between the formate oxygens along the *b* axis, shows alternating distances between H1⋯O3 and H1⋯O3a. There are certain zones in the structure where the H1⋯O3 atoms have a shorter separation, whereas at other points, H1⋯O3a has the shorter contact. This trend is observed at all three temperatures, driving the modulated phases. Compared with the parent compounds, there is no clear trend between the changes in the hydrogen bond distances and either the modulated structure that is adopted, or the composition of the compound [Fig. 6 ▸(*a*)].

The modulations are triggered by the hydrogen bond competition; however, the changes in the modulation vector can be observed by the resultant magnitude of displacement of the atom sites from the average structure. This can be followed through the *y* amplitude displacements of the metal site [Fig. 6 ▸(*b*)]. There is a division between displacements observed for the shorter modulation length (**q** ≃ 0.143c*, blue lines) and the atom displacements observed for the larger modulation length (**q** ≃ 0.124c*, red lines). This is more quantitatively conveyed in amplitude displacements for the sine term of the first-order harmonics in the Fourier series along *y* for the metal atoms (Table 3 ▸). At 70 K, at which temperature **2** has the modulation vector **q** = 0.1429 (2)c*, *y* = 0.02033 (15), which is similar to the displacement observed for Co, *y* = 0.0229 (106 K), and Ni analogues, *y* = 0.02274 (40 K) (Cañadillas-Delgado *et al.*, 2019, 2020 ▸). The phase transition to the longer modulation length [**q** = 0.1249 (2)c*] coincides with increasing amplitude displacements in the metal site: for **2***y* = 0.0322 (2) (30 K), compared with the Co analogue *y* = 0.0322 (5) (86 K). It is proposed that the Co analogue undergoes its final structural transition to a monoclinic non-modulated phase as the continual increases in the amplitude displacements with temperature eventually result in a division into two non-modulated domains (Canadillas-Delgado *et al.*, 2019 ▸). It is possible that the shorter Ni—O bond lengths limit the atom displacement, preventing larger atomic displacement values from being reached. The *M*—O bond distances at 30 K for **2** are intermediate of the Co—O and Ni—O values, as expected for a solid solution (Table 4 ▸) (Lee *et al.*, 2016 ▸; Shanmukaraj & Murugan, 2004 ▸). It is likely that this factor aids in dictating and limiting the phases accessible for each compound.

**Figure 7:**
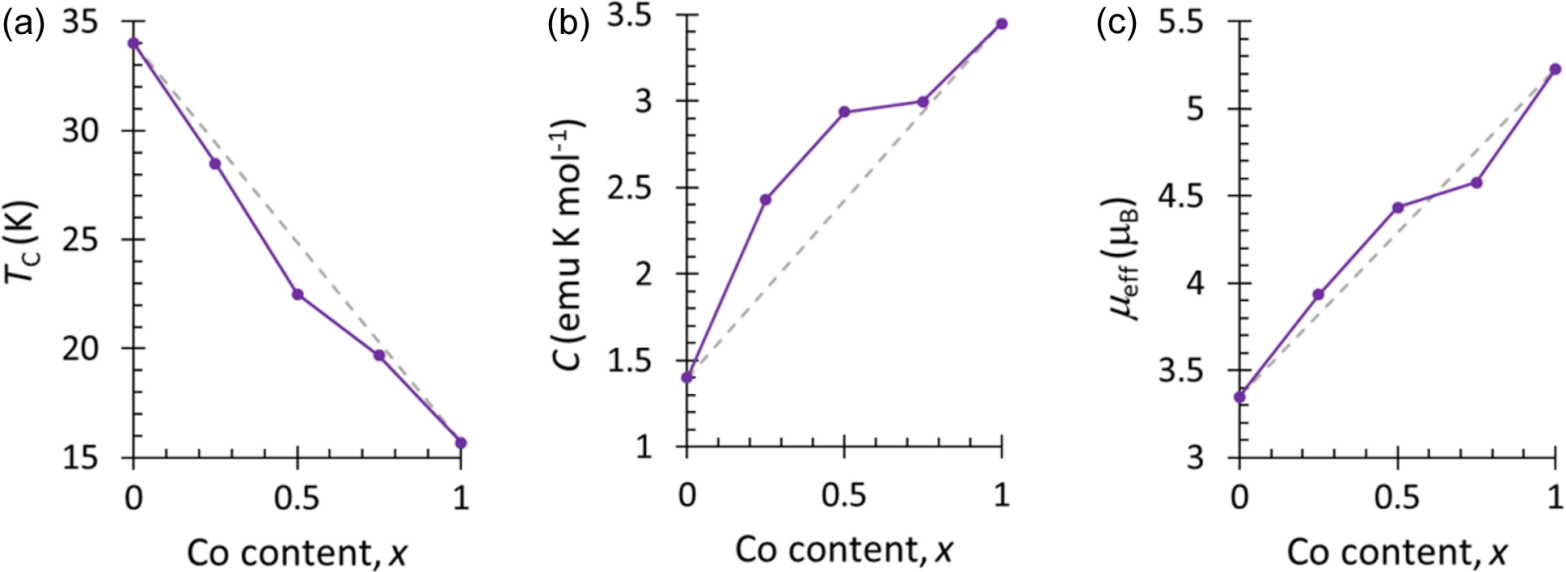
Summary of magnetometry values obtained from susceptibility measurements of [CH_3_NH_3_]Co*_x_*Ni_1−*x*_(HCOO)_3_ (*x* = 0, 0.25, 0.50, 0.75 and 1). (*a*) Magnetic ordering temperatures, *T*_C_. (*b*) Curie constant, *C*, extracted from the Curie–Weiss fit in the range 150–300 K for the solid solutions and in the ranges 20–300 and 50–300 K for pure Co and pure Ni compounds, respectively. (*c*) Effective magnetic moment from the Curie–Weiss fit, μ_eff_. Values for *x* = 0 and *x* = 1 are from their reported values (Pato-Doldán *et al.*, 2016 ▸; Gómez-Aguirre *et al.*, 2016 ▸). The dashed lines can be used as a guide for the eye between end members.

The bulk magnetic properties of **1**, **2** and **3** exhibit a continuous linear trend between the Ni and Co end members. This includes a decrease in the ordering temperature and an increase in the Curie constant and effective magnetic moment as the Co content decreases (Fig. 7 ▸ and Table S1). Cobalt–nickel solid solutions of molecular frameworks, such as dicyanamides (Lee *et al.*, 2016 ▸) and hypophosphites (Marcos *et al.*, 1993 ▸), report similar trends, with the ordering temperatures increasing almost linearly towards the Ni parent compound. These are rationalized by the strengthening of superexchange interactions as a result of decreasing *M*—O bond lengths with increasing Ni content (Lee *et al.*, 2016 ▸). It is plausible that this can explain the magnetic properties observed for these formate compounds as well, where the bond lengths for **2** follow this trend (Table 4 ▸). It is possible, however, to observe the strength of the antiferromagnetic correlations in the isothermal magnetization measurements. The degree of saturation at 5.00 (1) T (*M*_5T_/*M*_sat._) decreases from 0.24 for **3**, to 0.17 for **2** and 0.14 for **1**. The decrease in the extent of saturation suggests that, by increasing the Ni content, the antiferromagnetic correlations are strengthened.

Both Ni^2+^ and Co^2+^ ions have large orbital contributions to the magnetic moment, due to the second-order spin–orbit coupling for Ni^2+^ in an ^3^*A* term, and the unquenched orbital moment in the ^4^*T* term of Co^2+^. As these orbital components do not follow the classical Curie–Weiss dependence on temperature, θ_CW_ varied greatly with the temperature range used to perform the fit for all these formate compounds.

Compound **2** magnetically orders at *T*_C_ = 22.5 (7) K in the magnetic superspace group *Pn′ma′*(00γ)0*s*0 with **q** = 0.1249 (2)c*. The moments show weak ferromagnetic ordering, with the uncompensated moment along the *b* axis. This ordering is broadly comparable to both the Co and the Ni end members, which both present weak ferromagnetic superexchange with the nearest neighbours. Compound **2** orders in the same superspace group as the Ni analogue, although with a smaller modulation vector [**q** = 0.1426 (2)c* for [CH_3_NH_3_]­Ni(HCOO)_3_ compound]. Unlike the magnetic structure of the Ni compound, **2** adopts an improper modulated magnetic structure. It has been reported that by applying a small external magnetic field, approximately 0.05 T, [CH_3_NH_3_]­Ni(HCOO)_3_ undergoes a transition to an improper incommensurate magnetic phase with collinear moments (Pato-Doldán *et al.*, 2023 ▸). The activation of any proper magnetic modulations in the structure may be suppressed in **2** by the weaker superexchange pathways resulting from coupling of the Co ions or the modulation of the *M*—O—C bond angles, which might have a similar effect to the small external magnetic field in the Ni analogue.

## 4. Conclusions

Three solid solutions of [CH_3_NH_3_]Co*_x_*Ni_1−*x*_(HCOO)_3_, *x* = 0.25 (**1**), *x* = 0.50 (**2**) and *x* = 0.75 (**3**), have been synthesized and their nuclear structures and magnetic properties identified through single-crystal neutron diffraction and magnetization measurements. Magnetometry data reveal that their bulk magnetic properties exhibit a linear continuum between the Ni and Co end members. The Laue neutron diffraction data permitted a practical method to track the structural behaviour of the compounds and identify the temperature regions of the low-temperature modulated phases, with good estimation of the wavevectors.

Monochromatic neutron diffraction data reflect that, similar to the Ni and Co end members, the modulated phases for **2** are induced by the competing hydrogen bond interactions. However, the structural evolution does not follow the same phases as either parent compound. This is likely a result of the differing Co—O and Ni—O bond lengths which dictate the limits of the atom amplitude displacement modulations. A direct comparison with the pure Co compound reveals that the introduction of a solid solution directly affects the crystalline quality of the sample, as seen by the absence of third-order satellite reflections after the phase transition corresponding to the change of the wavevector from **q** = 0.1429 (2)c* to **q** = 0.1249 (2)c*.

These solid solutions have shown that, through doping of the metal site, the bulk magnetic properties – in particular the magnetic ordering temperature – of [CH_3_NH_3_]­Co*_x_*Ni_1−*x*_(HCOO)_3_ can be tuned through the metal ratios. In addition, both the transition temperatures and the nature of the nuclear phase transitions can be manipulated via the Ni content. Note that our results advocate that doping the samples increases frustration in the structure, leading to the stabilization of modulated structures over a broader temperature range. Moreover, our findings indicate that the energy barrier separating distinct structural phases is minimal, implying the feasibility of transitioning between them via external stimuli, such as pressure.

The study of modulated structures constitutes an important step in the better understanding of the structure–property relationship of CPs. Despite the sparsity of reported aperiodic molecular frameworks, with understanding of the interactions, this study presents the opportunity to consciously design molecular compounds with the propensity for modulated phases and finer control of their properties.

## 5. Related literature

The following references are cited in the supporting information: Bain & Berry (2008 ▸); Duisenberg (1992 ▸); Katcho *et al.* (2021 ▸); Matthewman *et al.* (1982 ▸); McIntyre & Stansfield (1988 ▸); Ouladdiaf *et al.* (2011 ▸); Palatinus & Chapuis (2007 ▸); Petříček *et al.* (2014 ▸); Rodríguez-Carvajal *et al.* (2018 ▸); Sheldrick, G. M. (2015*a* ▸,*b* ▸); Wilkinson *et al.* (1988 ▸).

## Supplementary Material

Crystal structure: contains datablock(s) comp2_70K, I_comp2_70K, comp2_30K, I_comp2_30K, comp2_2K, I_comp2_2K, comp2_RT, I_comp2_RT, comp1_RT, I_comp1_RT, comp3_RT, I_comp3_RT. DOI: 10.1107/S2052252524008583/lt5069sup1.cif

The magnetic structure of compound 2. DOI: 10.1107/S2052252524008583/lt5069sup2.txt

Supporting figures and tables. DOI: 10.1107/S2052252524008583/lt5069sup3.pdf

CCDC references: 2351889, 2351890, 2351891, 2380908, 2380909, 2380910

## Figures and Tables

**Table 1:** Experimental and crystallographic data for compounds **1**–**3**, measured on D9 and D19 neutron diffractometers refined with the *JANA2020* software (Petříček *et al.*, 2023 ▸) All hydrogen atom parameters were refined for all compounds. RT: room temperature.

Compound	**1**	**2**	**2**	**2**	**2**	**3**
Chemical formula	C_4_H_9_Co*_x_*Ni_1−*x*_NO_6_					
Refined *x*	0.297 (9)	0.526 (8)	0.526 (8)	0.526 (8)	0.526 (8)	0.765 (2)
*M*_r_	225.9	225.9	225.9	225.9	225.9	226.0
*Z*	4	4	4	4	4	4
Diffractometer	D9	D9	D19	D19	D19	D9
Temperature (K)	RT	RT	70	30	2	RT
Space group	*Pnma*	*Pnma*	*Pnma*(00γ)0*s*0	*Pnma*(00γ)0*s*0	*Pn*′*ma*′(00γ)0*s*0	*Pnma*
*a* (Å)	8.358 (2)	8.3506 (4)	8.2052 (2)	8.2003 (2)	8.2010 (3)	8.372 (2)
*b* (Å)	11.637 (3)	11.6556 (8)	11.5759 (3)	11.5737 (3)	11.5747 (8)	11.705 (4)
*c* (Å)	8.069 (2)	8.0831 (4)	8.1141 (2)	8.1133 (2)	8.1144 (3)	8.095 (2)
*V* (Å^3^)	784.8 (3)	786.74 (8)	770.70 (3)	770.02 (3)	770.25 (7)	793.2 (4)
Wavevectors	–	–	**q** = 0.1429 (2)c*	**q** = 0.1249 (2)c*	**q** = 0.1249 (2)c*	–
ρ_calc_ (mg m^−3^)	1.9118	1.9075	1.9472	1.9489	1.9483	1.8925
λ (Å)	0.8348	0.8359	1.45567	1.45567	1.45567	0.8359
μ (mm^−1^)	0.005	0.006	0.011	0.011	0.011	0.008
*R*_1_, *I* > 3σ(*I*) (all)	0.0354 (0.0574)	0.0361 (0.0611)	0.0601 (0.0762)	0.1189 (0.1315)	0.1217 (0.1343)	0.0636 (0.1109)
*wR*_2_, *I* > 3σ(*I*) (all)	0.1048 (0.1067)	0.0531 (0.0645)	0.2185 (0.2321)	0.1899 (0.2034)	0.1802 (0.1901)	0.1235 (0.1525)
No. of parameters	107	107	164	449	452	107
Independent reflections	737	1178	3291	3346	3346	528
No. of main reflections	–	–	682	694	694	–
No. of first-order satellite reflections	–	–	1230	1250	1250	–
No. of second-order satellite reflections	–	–	1379	1402	1402	–

**Table 2:** Amplitude displacements for the sine term of the first-order harmonics in the Fourier series of the metal site for compound **2**

	70 K	30 K	2 K
*x*	0.00216 (13)	0.00283 (19)	0.00235 (19)
*y*	0.02033 (15)	0.0322 (2)	0.0335 (2)
*z*	0.00009 (14)	0.00020 (19)	0.0001 (2)

**Table 3:** Amplitude displacements along *y* of the metal site for [CH_3_NH_3_]Co(HCOO)_3_, compound **2** and [CH_3_NH_3_]Ni(HCOO)_3_ for different modulation vectors

Compound	**q**	*y*	*T* (K)	**q**	*y*	*T* (K)
[CH_3_NH_3_]Co(HCOO)_3_†	0.1430 (2)	0.0229 (4)	106	0.1247 (2)	0.0322 (5)	86
**2**	0.1429 (2)	0.02033 (15)	70	0.1249 (2)	0.0322 (2)	30
[CH_3_NH_3_]Ni(HCOO)_3_†	0.1426 (2)	0.02274 (11)	40			

† Values for Co and Ni compounds obtained from Canadillas-Delgado *et al.* (2019 ▸) and Cañadillas-Delgado *et al.* (2020 ▸), respectively.

**Table 4:** Average bond length comparison of the metal environment for [CH_3_NH_3_]Co(HCOO)_3_, compound **2** and [CH_3_NH_3_]Ni(HCOO)_3_ at 45, 30 and 40 K, respectively

Compound	*M*—O1 (Å)	*M*—O2 (Å)	*M*—O3 (Å)
[CH_3_NH_3_]Co(HCOO)_3_†	2.083 (3)	2.101 (3)	2.090 (3)
**2**	2.070 (2)	2.087 (2)	2.078 (4)
[CH_3_NH_3_]Ni(HCOO)_3_†	2.0555 (19)	2.069 (2)	2.059 (4)

† Values for Co and Ni compounds obtained from Mazzuca *et al.* (2018 ▸) and Cañadillas-Delgado *et al.* (2020 ▸), respectively.
