# Supplementary material for: Tuning structural modulation and magnetic properties in metal–organic coordination polymers [CH3NH3]CoxNi1−x(HCOO)3
Source: IUCrJ. 2024 Sep 24;11(Pt 6):910–20. doi: 10.1107/S2052252524008583 (PMC11533998; doi:10.1107/S2052252524008583)
Supplement: Supplementary file 3 [file m-11-00910-sup3.pdf]

IUCrJ

Volume 11 (2024)

Supporting information for article:

Tuning Structural Modulation and Magnetic Properties in Metal-Organic Coordination Polymers [CH3NH3]CoxNi1−x(HCOO)3

Madeleine Geers, Oscar Fabelo, Matthew J. Cliffe and Laura Cañadillas-Delgado

Crystallographic data, in CIF format, for the structures of **2** collected at 70, and 30 K have been deposited at The Bilbao Incommensurate Crystal Structure Database with entry codes Bn1PFN5BD2w and 4YKOEFilc1S, respectively. The crystallographic data, in CIF format, for the commensurate structures at RT of **1**, **2** and **3** can be downloaded from the Cambridge Crystallographic Data Centre through the CCDC reference numbers from 2351889 to 2351891. The magnetic structure of compound **2** at 2 K, in magCIF format, has been deposited at The Magndata Bilbao Structure Database with entry code xxxxxx.

S1. Experimental

S1.1. Synthesis

The synthesis of $[CH_3NH_3]Co_xNi_{1-x}(HCOO)_3$ $x = 0.25$ (**1**), 0.5 (**2**) and 0.75 (**3**) compounds were followed from reported methods for protonated amine metal formates (Mazzuca *et al.*, 2018; Wang, *et al.*, 2004). An example (compound **2**) is provided for quantities and timescales used for the synthesis.

Aqueous solutions of $CoCl_2 \cdot 6H_2O$ (119 mg, 0.5 mmol, 1.5 mL), $NiCl_2 \cdot 6H_2O$ (119 mg, 0.5 mmol, 1.5 mL), CH_3NH_3Cl (68 mg, 1 mmol, 3 mL) and NaHCOO (204 mg, 3 mmol, 2 mL) were mixed with *N*-methylformamide ($HCONHCH_3$). The solution was sealed in an autoclave (43 mL) and heated at 413 K for 3 days. The solution was slowly cooled to ambient temperature (approximately 5 h), yielding dark green prismatic crystals ($2.3\times1.6\times1.2$ mm^3). The crystals were filtered, and dried at room temperature.

Using an analogous method with stoichiometric ratios of $CoCl_2 \cdot 6H_2O$ and $NiCl_2 \cdot 6H_2O$ yield dark green crystals of **1** ($2\times1.5\times1.5$ mm^3) and dark red crystals of **3** ($1.5\times1.5\times1$ mm^3).

S1.2. Magnetic measurements

Measurements of the magnetic susceptibility were carried out on samples of compounds **1** (12.45 mg), **2** (12.50 mg) and **3** (8.75 mg) using a Quantum Design Magnetic Property Measurements System (MPMS) and a Superconducting Quantum Interference Device (SQUID) magnetometer. Field-cooled (FC) and zero-field-cooled susceptibility measurements were carried out on microcrystalline samples using an applied field of 100 Oe The zero-field-cooled (ZFC) and field-cooled (FC) susceptibility were measured in an applied field of 0.01 T over the temperature range 2 − 300 K. As $M(H)$ is linear in this field, the small-field approximation for the susceptibility, $\chi(\mathrm{T}) \doteq \frac{M}{H}$, where M is the magnetisation and H is the magnetic field intensity, was taken to be valid. Isothermal magnetisation measurements were carried out at 2 K over the field range −5 to +5 T. Data were corrected for diamagnetism of the sample using Pascal's constants (Bain *et al.*, 2008).

S1.3. Laue neutron diffraction

The Laue neutron diffraction measurements were collected on the multiple CCD diffractometer CYCLOPS (Cylindrical CCD Laue Octagonal Photo Scintillator, at the ILL, France) which operates with thermal neutrons (Ouladdiaf *et al.*, 2011). Single crystals of 2 × 1.5 × 1.5 mm^3 $[CH_3NH_3]Co_{0.25}Ni_{0.75}(HCOO)_3$ (**1**), 2.6 × 1.6 × 1.2 mm^3 $[CH_3NH_3]Co_{0.50}Ni_{0.50}(HCOO)_3$ (**2**) and 1.5 × 1.5 × 1 mm^3 $[CH_3NH_3]Co_{0.75}Ni_{0.25}(HCOO)_3$ (**3**), were mounted on a vanadium pin and placed in a standard orange cryostat. The diffraction patterns were recorded in the temperature range 10 to 120 K, following a ramp of 0.1 K per 30 s. Each Laue diffraction pattern was collected over a period of 15 min with a temperature range of 3 K. The samples were centred on the neutron beam by maximisation of the intensity of several strong reflections in the x, y and z directions, after which, a specific orientation was selected and the temperature evolution was collected. Graphical visualisation of the Laue patterns was performed with the ESMERALDA software (Rodríguez-Carvajal *et al.*, 2018). Because the neutron beam comprises the wavelength range from 0.8 to 3.2 Å (thermal neutrons) the peaks highlighted in figures S5, S6 and S7 correspond to multiple spots, i.e. there is more than one reflection at different wavelengths contributing to the peak.

S1.4. Ambient temperature single crystal neutron diffraction (D9)

Hot neutron single crystal diffraction was performed on the four-circle diffractometer D9 (ILL, France) for compounds **1**, **2** and **3**. A monochromatic beam of wavelength $\lambda = 0.836$ Å was produced using the (220) plane of a Cu crystal in transmission geometry, and a small two-dimensional area detector. Integrated intensities of structural Bragg peaks were collected with standard transverse scans (ω-scans). NOMAD software from the ILL was used for data collection. The structural models were solved using the SUPERFLIP program (Palatinus *et al.*, 2007) and refined using Jana2020 (Petříček *et al.*, 2023), refining the occupancies of the metal site to obtain the cobalt and nickel ratio of each crystal.

S1.5. Low temperature single crystal neutron diffraction (D19)

Monochromatic single crystal neutron diffraction data were collected on the four-circle D19 diffractometer (ILL, France) for compound **2**. Neutrons with a wavelength of 1.456 Å were provided by a flat Cu monochromator using the 220 reflection at $2\theta_M = 69.91°$ take-off angle. The sample was placed in a closed-circuit displex cooling device, which was operated following a ramp of 2 K min^{-1}. Measurements were taken at 2 K, and at 10 K intervals between 30 and 100 K. NOMAD software from the ILL was used for data collection. Longer data acquisitions were made at 2, 30 and 70 K and were used for the nuclear and magnetic refinements. Unit cell determinations were performed using PFIND and DIRAX programs, and processing of the raw data was applied using RETREAT, RAFD19 and Int3D programs (Duisenberg, 1992; McIntyre *et al.*, 1988; Wilkinson *et al.*, 1988 & Katcho *et al.*, 2021). The data were corrected for the absorption of the low-temperature device using

the D19ABSCAN program (Matthewman *et al*, 1982). Nuclear and magnetic models were solved by using the SUPERFLIP program and refined using Jana2020 (Petříček *et al*., 2023). The amplitudes of the first and second order harmonics in the Fourier series of each parameter were refined for all atoms. After the refinement, all those second-order harmonics with values equal to zero were removed in order to avoid overparameterisation. The modulation of the magnetic moments of the metal atoms in the 2 K data set was refined, but the resulting values were close to zero and the statistics did not improve. Consequently, these parameters were removed.

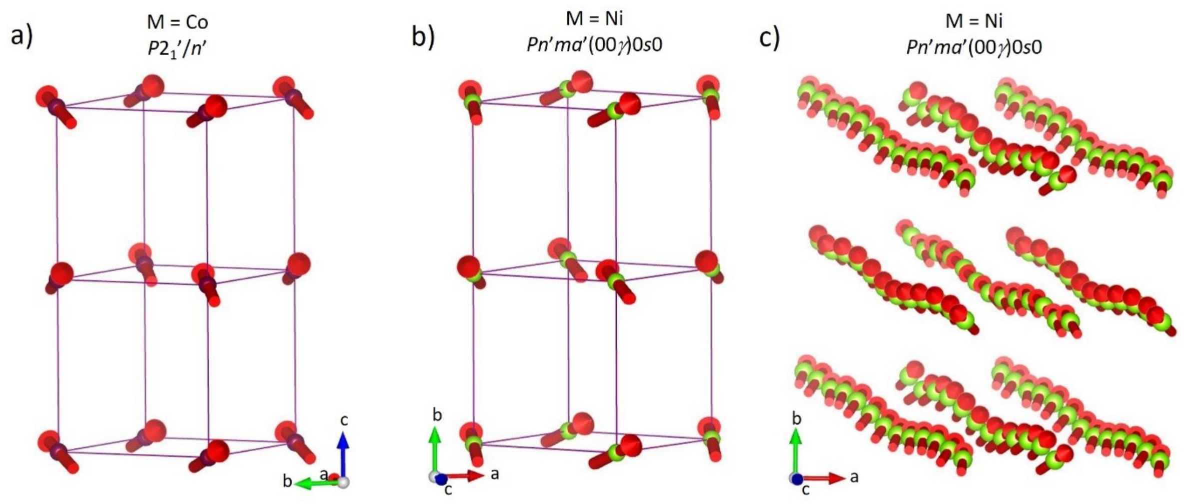

Figure S1 The magnetic structures for $[CH_3NH_3]M(HCOO)_3$. Both compounds magnetically order with weak ferromagnetic interactions. a) $[CH_3NH_3]Co(HCOO)_3$ in the non-modulated, monoclinic $P2'_1/n'$ magnetic space group, measured at 2 K. b) The average magnetic structure of $[CH_3NH_3]Ni(HCOO)_3$ ordering in the incommensurately modulated $Pn'ma'(00\gamma)0s0$ magnetic superspace group, measured at 5 K. The nearest neighbour interactions are displayed with purple lines. c) Modulated magnetic structure of the $[CH_3NH_3]Ni(HCOO)_3$ compound showing the modulation of the atoms, displaced predominately along the *b* axis, and the modulation of the magnetic moments with oscillations in the *ac* plane. The graphical representation has been carried out taking into consideration a super-cell that is ten times of the average structure along the *c* axis in order to include at least a full period.

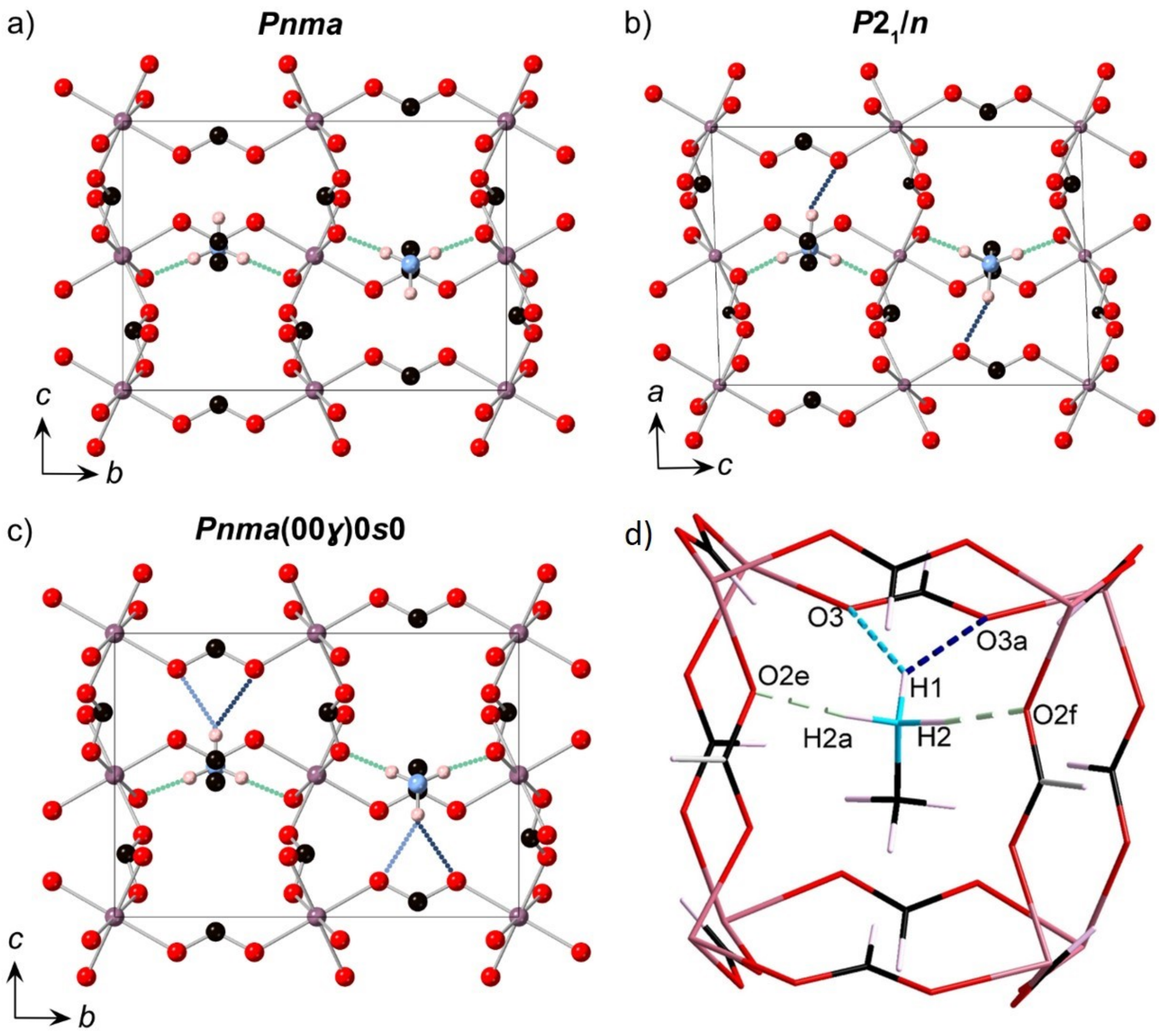

Figure S2 Schematic view of the possible hydrogen bonds in $[CH_3NH_3]M(HCOO)_3$ M = Co, Ni where green and blue dotted lines indicate the contacts involving H2 and H1 atoms from the NH_3 group, respectively. a) For the non-modulated *Pnma* phase at ambient temperature. b) The low temperature, non-modulated $P2_1/n$ phase obtained for M = Co below $T = 78$ K (Mazzuca *et al*, 2018). c) and d) The average structure of the incommensurately modulated phases *Pnma*(00*γ*)0*s*0, where blue dotted lines highlight the alternated H-bond interactions involving H1 (Cañadillas-Delgado *et al*, 2019). Metal = purple, O = red, C=black, N= blue, H = pale pink.

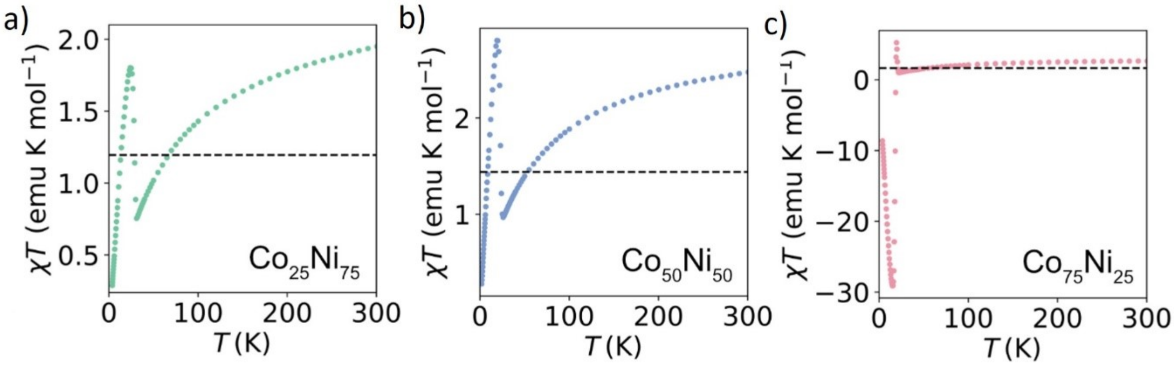

Figure S3 Plots of the variable temperature magnetic susceptibility product, with the high temperature spin only Curie value, $C_{\text{spin only}}$, indicated with a dashed line. a) Compound **1**, $C_{\text{spin only}}$ = 1.21 emu K mol^{-1}, b) Compound **2**, $C_{\text{spin only}}$ = 1.44 emu K mol^{-1} and c) Compound **3**, $C_{\text{spin only}}$ = 1.66 emu K mol^{-1}.

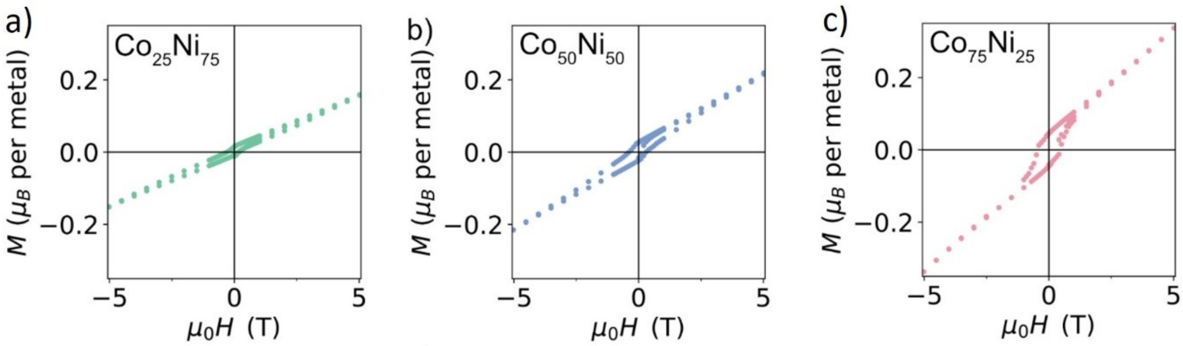

Figure S4 Isothermal magnetisation measurements for compounds **1** (a), **2** (b) and **3** (c) measured at 2 K between −5.00(1) and 5.00(1) T.

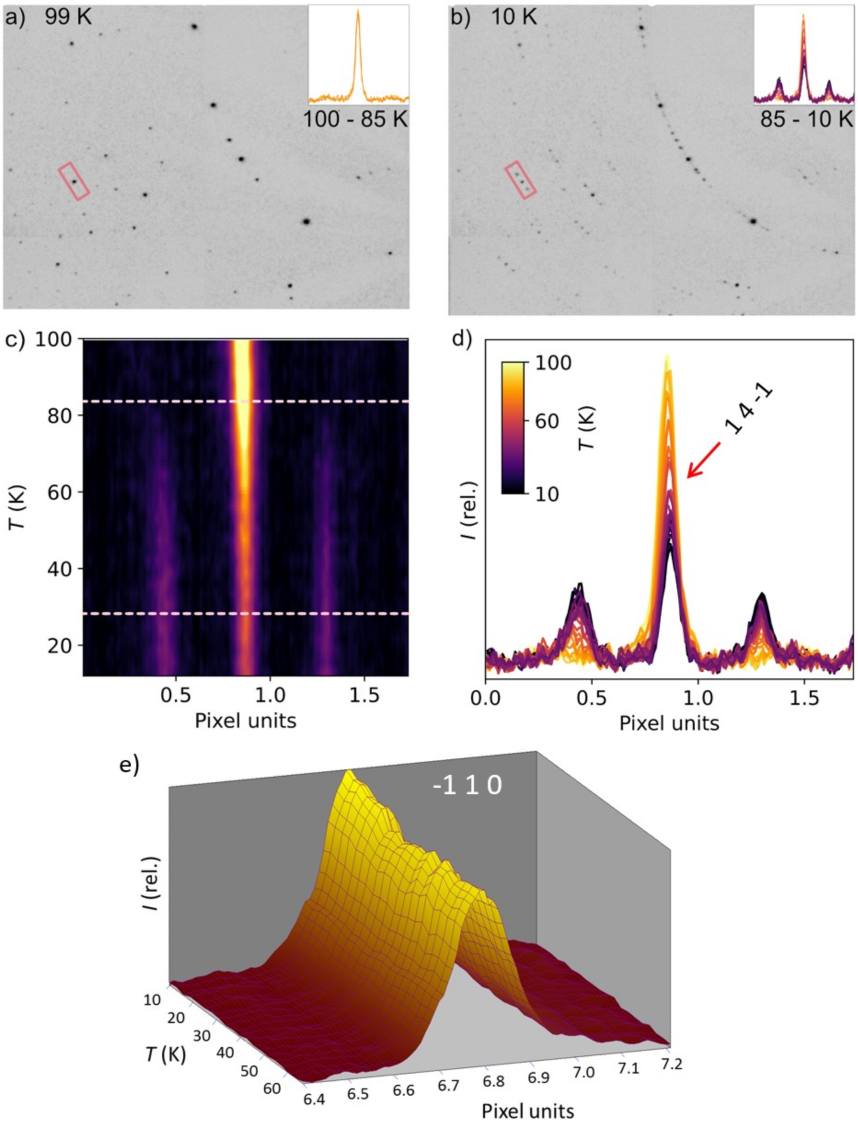

Figure S5 Laue neutron diffractograms (CYCLOPS, ILL), heating with a ramp of 3 K per image collected during 15 min. for compound **1**. The insets (in a and b) display the integrated intensity for the pixels in the box highlighted in pink, following the multiple spot that contains the 1 4 -1 reflection over the temperature ranges specified. The diffractograms were measured at a) 99 K, showing the non-modulated phase. b) At 10 K showing the modulated phase. c) Temperature evolution in the range 10−100 K for the pixel intensity in the pink box following the multiple spot containing the 1 4 -1 reflection. The pink dashed lines indicate the approximate temperature of the phase transition (structural at 85(3) K, and magnetic, obtained from magnetometry data, at 28.5(5) K. d) Superposition of all the integrated intensities from the insets (a and b) over the temperature range 10−100 K. In c) and d) the multiple spot containing the main reflection 1 4 -1 and the multiple spots corresponding to satellite reflections are observed, on both sides of the main reflection, below about 85(3) K. e) Temperature evolution of the multiple spot containing the -110 reflection, where an increase of intensity at about 28(3) K is observed. The -110 is a structurally forbidden reflection that also increase its intensity because of magnetic order in the pure nickel compound (Cañadillas-Delgado *et al*., 2020).

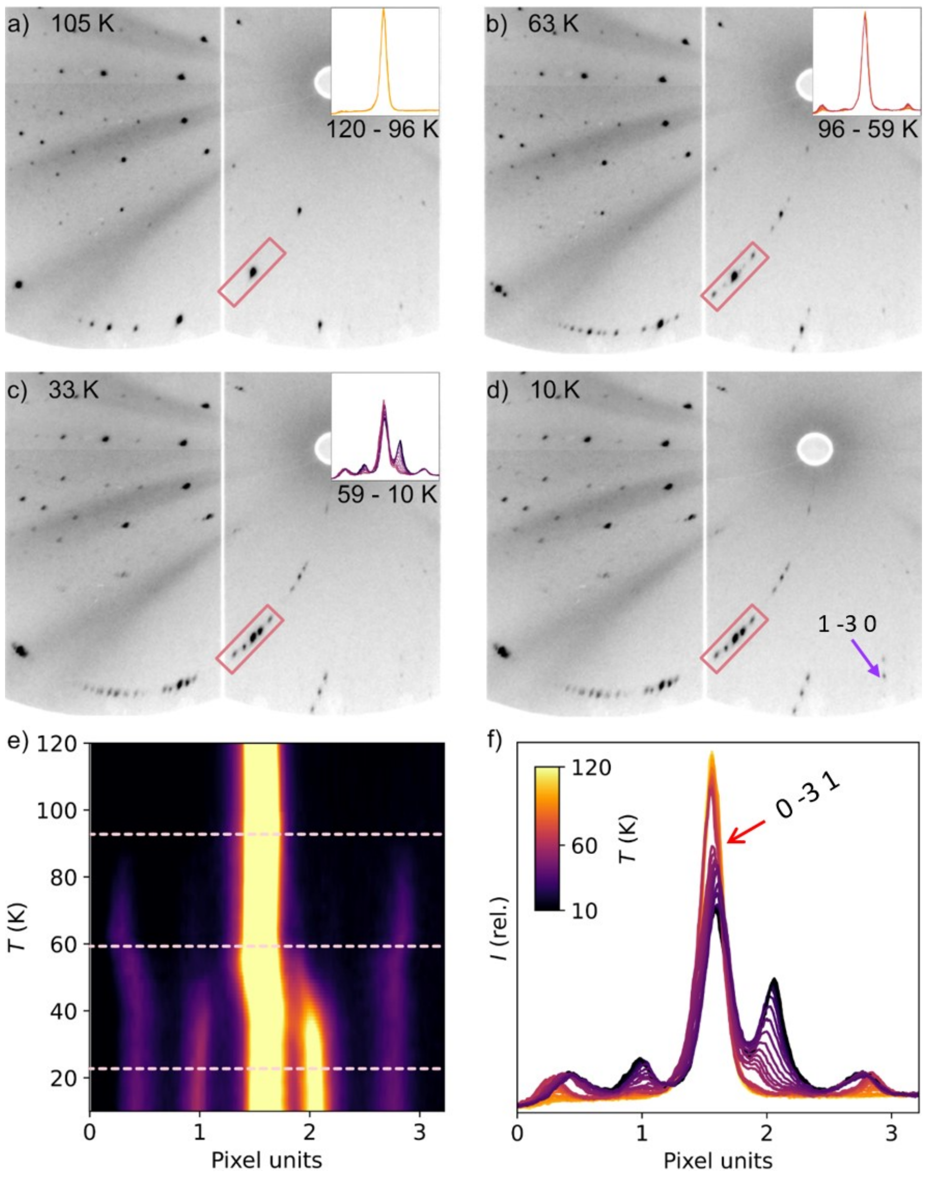

Figure S6 Laue neutron diffractograms (CYCLOPS, ILL), heating with a ramp of 3 K per image collected during 15 min. for compound **2**. The insets (in a, b and c) display the integrated intensity for the pixels in the box highlighted in pink, following the multiple spot containing the 0 3 -1 reflection over the temperature ranges specified. The diffractograms were measured at a) 105 K, showing the non-modulated phase, b) at 63 K, and c) at 33 K, showing the first and second modulated phases, respectively, and d) at 10 K where a visible magnetic reflection (multiple spot containing the 1 -3 0 reflection) has been highlighted with a purple arrow. e) Temperature evolution over the range 10−120 K for the pixel intensities in the pink box. The pink dashed lines highlight the approximate temperature of the phase transitions (structural at 96(3) K and 59 (3) K and magnetic at 22.5(7) K obtained from magnetometry data). f) Superposition of all the integrated intensities from the insets (a, b and c) over the temperature range 10−120 K. In e) and f) the multiple spot containing the main reflection 0 -3 1 with the multiple spots corresponding to satellite reflections, on both sides of the main reflection, are observed below 96(3) K, and change of distances below 59(3) K.

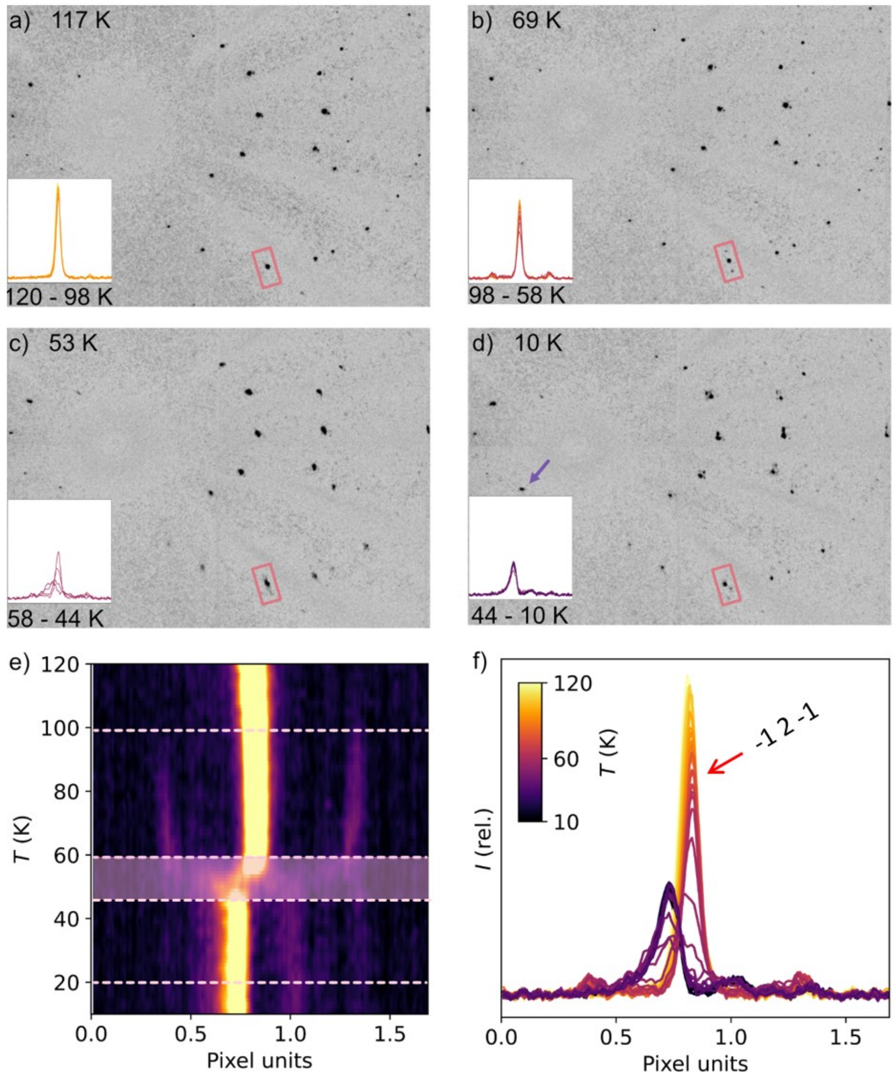

Figure S7 Laue neutron diffractograms (CYCLOPS, ILL) for compound **3**, following a heating ramp of 3 K per image collected during 15 min. The insets (in a, b, c and d) display the integrated intensity for the pixels in the box highlighted in pink, following the multiple spot containing the -1 2 -1 (*Pnma*) reflection over the temperature ranges specified. The diffractograms were measured at a) 117 K, showing the non-modulated phase, b) at 69 K, showing the modulated phase with weak satellite reflections visible, c) at 53 K, showing the slow phase transition, and d) at 10 K, showing the twinned, non-modulated $P2_1/n$ phase. In d) a magnetic reflection is indicated with a purple arrow. e) Temperature evolution over the 10−120 K range for the pixel intensities in the pink box. The pink dashed lines indicate the approximate temperature of the structural phase transition at 98(3) K, the magnetic order at 19.7(5) K, from the magnetometry data, and the highlighted pink region shows the slow phase transition between 58(3) and 44(3) K. f) Superposition of all the integrated intensities from the insets (a, b, c and d) over the temperature range 10−120 K. In e) and f) the multiple spot containing the main reflection -1 2 -1 with the multiple spots corresponding to satellite reflections, on both sides of the main reflection, are observed between 98(3) K and 44(3) K, with a slight change in

the distance between the main and satellite reflections below 58(3) K. Below 44(3) K it could be seen that the pattern changes drastically indicating the appearance of the non-modulated monoclinic twined phase.

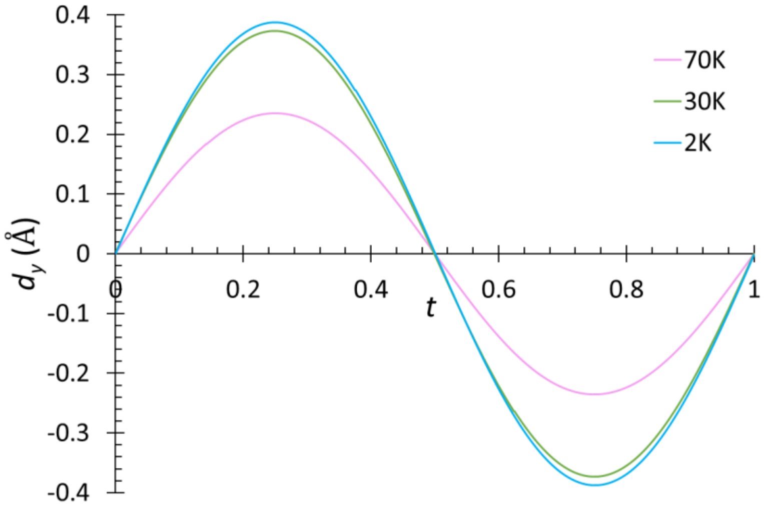

Figure S8 The modulation function of the metal site exhibiting the displacement along y, d_y, observed from the refinements of compound **2** measured using the D19 diffractometer (ILL) at 2 K (blue line), 30 K (green line) and 70 K (pink line).

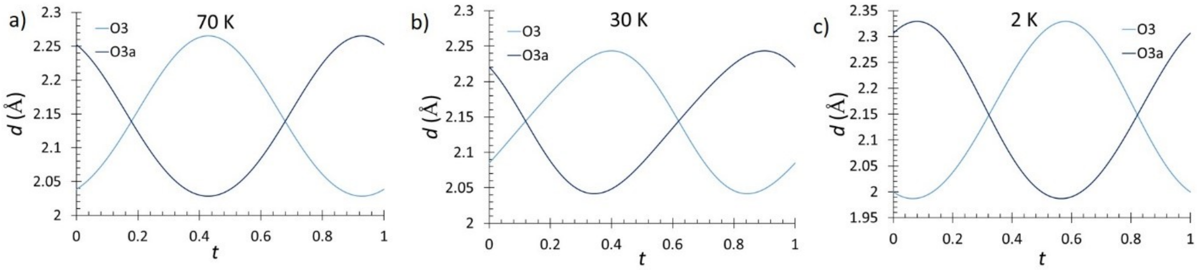

Figure S9 Modulation of the H1···O3 distances for compound **2** at a) 70 K, b) 30 K and c) 2 K.

Table S1 Summary of the bulk magnetic properties for $[CH_3NH_3]Co_xNi_{1-x}(HCOO)_3$ $x = 0, 0.25, 0.50, 0.75$ and 1, obtained from magnetisation measurements. C is calculated from the value of χT at 300 K. The θ_{CW} values are calculated from fits over the temperature ranges $150 < T < 300$ K.

x	0*	0.25	0.5	0.75	1**
S	1.0	1.125	1.25	1.375	1.5
T_C (K)	34	28.5(5)	22.5(7)	19.7(5)	15.9
θ_{CW} (K)	−64.9	−70.8(7)	−56.3(1)	−43.6(5)	−43.5
C (cm^3 K mol^{-1})	1.40	1.936(4)	2.461(1)	2.625(1)	3.45
$C_{spin\ only}$ (cm^3 K mol^{-1})	1.0	1.21	1.44	1.66	1.87
$\mu_{eff.}$ (μ_B)	3.3	3.936(2)	4.436(2)	4.578(2)	5.23
$\mu_{spin\ only}$ (μ_B)	2.83	3.09	3.35	3.61	3.88
$M_{rem.}$ (μ_B per metal)		0.015(1)	0.027(1)	0.045(3)	
H_C (T)		0.15(1)	0.30(1)	0.45(1)	
$M_{5\ T}/M_{sat.}$		0.140	0.175	0.245	

* Values extracted from Pato-Doldán *et al.*, 2016.

** Values extracted from Gómez-Aguirre *et al.*, 2016.

Table S2 Bond lengths distances of the metal environment obtained from the refinement of compound **2** at 70 K, 30 K and 2 K, where it could be appreciated the largest displacement in M-O1 and M-O3 distances at 30 K.

T (K)	Bond	Average (Å)	Maximum (Å)	Minimum (Å)	Max. displacement (Å)
70	M-O1	2.0696(16)	2.0736(19)	2.0658(19)	+0.004(2), -0.0038(19)
	M-O2c	2.0835(15)	2.0879(18)	2.0793(18)	+0.0044(18), -0.0042(18)
	M-O3	2.0779(18)	2.081(2)	2.075(2)	+0.003(2), -0.003(2)
30	M-O1	2.070(2)	2.130(3)	2.014(3)	+0.060(3), -0.056(3)
	M-O2c	2.087(2)	2.094(3)	2.081(3)	+0.007(3), -0.006(3)
	M-O3	2.078(4)	2.138(5)	2.009(5)	+0.060(5), -0.069(5)
2	M-O1	2.069(2)	2.077(3)	2.061(3)	+0.008(3), -0.008(3)
	M-O2c	2.086(2)	2.096(3)	2.077(3)	+0.010(3), -0.009(3)
	M-O3	2.075(5)	2.090(5)	2.066(5)	+0.015(5), -0.009(5)

Symmetry code: $c = -x+1/2, -y+1, z+1/2$
